# Identification of candidate tolerance genes to low-temperature during maize germination by GWAS and RNA-seqapproaches

**DOI:** 10.1186/s12870-020-02543-9

**Published:** 2020-07-14

**Authors:** Hong Zhang, Jiayue Zhang, Qingyu Xu, Dandan Wang, Hong Di, Jun Huang, Xiuwei Yang, Zhoufei Wang, Lin Zhang, Ling Dong, Zhenhua Wang, Yu Zhou

**Affiliations:** 1 Key Laboratory of Germplasm Enhancement, Physiology and Ecology of Food Crops in Cold Region, College of Agronomy, Northeast Agricultural University, Harbin, 150030 Heilongjiang China; 2 Guangdong Provincial Key Laboratory of Plant Molecular Breeding, South China Agricultural University, Guangzhou, 510642 Guangdong China

**Keywords:** Maize, Low-temperature, Germination, Genome-wide association study, Candidate genes

## Abstract

**Background:**

Maize (*Zea mays* L.) is one of the main agricultural crops with the largest yield and acreage in the world. However, maize germplasm is very sensitive to low temperatures, mainly during germination, and low temperatures significantly affect plant growth and crop yield. Therefore, the identification of genes capable of increasing tolerance to low temperature has become necessary.

**Results:**

In this study, fourteen phenotypic traits related to seed germination were used to assess the genetic diversity of maize through genome-wide association study (GWAS). A total of 30 single-nucleotide polymorphisms (SNPs) linked to low-temperature tolerance were detected (−log10(*P*) > 4), fourteen candidate genes were found to be directly related to the SNPs, further additional 68 genes were identified when the screen was extended to include a linkage disequilibrium (LD) decay distance of *r*^2^ ≥ 0.2 from the SNPs. RNA-sequencing (RNA-seq) analysis was then used to confirm the linkage between the candidate gene and low-temperature tolerance. A total of ten differentially expressed genes (DEGs) (|log_2_ fold change (FC)| ≥ 0.585, *P* < 0.05) were found within the set distance of LD decay (*r*^2^ ≥ 0.2). Among these genes, the expression of six DEGs was verified using qRT-PCR. *Zm00001d039219* and *Zm00001d034319* were putatively involved in ‘mitogen activated protein kinase (MAPK) signal transduction’ and ‘fatty acid metabolic process’, respectively, based on Gene Ontology (GO) and Kyoto Encyclopedia of Genes and Genomes (KEGG) enrichment analyses. Thus, these genes appeared to be related to low-temperature signal transduction and cell membrane fluidity.

**Conclusion:**

Overall, by integrating the results of our GWAS and DEG analysis of low-temperature tolerance during germination in maize, we were able to identify a total of 30 SNPs and 82 related candidate genes, including 10 DEGs, two of which were involved in the response to tolerance to low temperature. Functional analysis will provide valuable information for understanding the genetic mechanism of low-temperature tolerance during germination in maize.

## Background

Maize (*Zea mays* L.) originated from tropical and subtropical regions and has a relatively high temperature threshold for germination [1]. As a consequence, maize is inherently sensitive to low temperatures [2], particularly during germination, and thus is seldom cultivated at higher latitudes or in mountainous regions. When maize is cultivated in cold zones, the plants grow more slowly and have a relatively short growing season, which leads to lower seedling vitality and reduced yields [3, 4]. It is known that the minimum temperature for maize seed germination is approximately 10 °C. When the temperature drops to 6 to 8 °C, irreversible damage to cells and tissues occurs. Under these conditions, seeds normally will not germinate, and the growth of seedlings will stop [1]. Thus, 10 °C is normally chosen as the growth temperature for the identification and screening of proper maize germplasm [5]. In recent years, the occurrence of cold weather has become more uncertain, and low temperature has been occurring more frequently, especially during the germination period and early developmental stage of seedlings. Low-temperature stress not only decrease the both emergence rate of maize seeds and seedling vigor but also increases the chance of pathogenic infection by soil bacteria, which can severely reduce maize yields. Therefore, it is urgent to identify the gene(s) that provide low-temperature-tolerant germination in maize.

At present, significant progress has been made in the identification of genetic loci associated with low-temperature tolerance in maize. Their corresponding genes are normally identified by gene mapping and GWAS. Some indices, such as the emergence rate, seedling emergence index, seedling dry weight, relative average germination time, and percentage of relative viability, have been used for genetic loci identification in maize inbred lines and populations [6, 7]. For instance, a major QTL for low-temperature tolerance of photosynthesis was detected on chromosome 6 using the ETH-DL3 × ETH-DH7 population [8]. Two other QTLs on chromosomes 3.01 and 6.03 were reported, which affect leaf color at low temperatures, and a candidate gene, *luteus11*, was identified [9]. By the use of a panel of European flint maize inbred lines, 47 lines were found to harbor favorable alleles for six significant QTLs [10]. In another study, 12 QTLs controlling the low-temperature germination rate and primary radicle length were detected on chromosomes 4, 5, 6, 7 and 9 by the use of 243 lines of the intermated B73 × Mo17 (IBM) Syn4 recombinant inbred line (RIL) population [11]. Later, researchers identified 43 QTLs that explained 0.62% ~ 39.44% of the phenotypic variance for low-temperature seed germination in maize by the use of three connected F_2:3_ populations with inbred lines of two low-temperature-tolerant inbred lines, 220 and P9–10, and two susceptible lines, Y1518 and PH4CV [5]. Several SNPs associated with low-temperature-tolerance traits at the seedling and seed germination stages were detected using GWAS. For example, 43 SNPs associated with 10 low-temperature-tolerance traits in maize seedlings or seed germination were found, although no overlapping SNPs were identified at either developmental stage [12]. A total of 19 markers related to low-temperature tolerance were identified through GWAS of 375 inbred lines in the field and in growth chambers; the loci at these markers explained 5.7% ~ 52.5% of the phenotypic genetic variation at the seedling growth stage and chlorophyll fluorescence parameters [13]. A further GWAS revealed 18 candidate genes from 17 genetic loci associated with low-temperature-tolerant germination, where 10 candidate genes were supported by previous QTL studies [14].

Numerous low-temperature-tolerance genes have been identified in many crop species, and their underlying molecular mechanisms have been studied. In rice, *COLD1* interacts with the G-protein alpha-subunit, which in turn promotes GTPase activity, resulting in the activation of Ca^2+^ channels for low-temperature-induced responses [15]. During the reproductive growth period of rice, the protein kinase gene *CTB4a* is involved in maintaining high pollen fertility under low-temperature conditions, which increases the seed setting rate and yield. In addition, *CTB4a* interacts with *AtpB* to regulate the ATP content under low temperatures to enhance low-temperature tolerance [16]. The MADS-box family transcription factor OsMADS57 interacts with OsTB1 to regulate low-temperature tolerance in rice and helps balance growth and defense responses, which are dependent on *OsWRKY94*, their common target gene [17]. In addition to transcriptional control, phosphorylation of the Basic Transcription Factor 3 Like (BTF3L) protein by the protein kinase OST1 promotes the interaction between BTF3L and CBFs, which increases the stability of CBF proteins and enhances plant tolerance to low temperatures [18]. Similarly, numerous studies investigating the mechanisms of low-temperature tolerance in maize have been reported, but they have had a limited impact on maize breeding. Several maize genes, including *ZmCDPK1*, *ZmSEC14p* and *ZmMPK5*, with roles in low-temperature tolerance have been identified [19–22]. Some genes that control kernel weight or kernel number at low temperatures have also been cloned, including genes that were shown to induce the expression of bZIP-type and ERF/AP2-type transcription factors [23, 24].

There have been several studies on the low-temperature tolerance of maize during germination, but detailed evaluation indices are lacking. Thus, we established a standard evaluation system in the present study for genetic mapping, with particular consideration for the actual sowing conditions that occur during early spring in Northeast China. Low-temperature evaluation methods for maize often include both field-based and indoor identification methods. Compared with the indoor methods, the field-based methods are more objective and can be used to evaluate the low-temperature tolerance of maize during each growth period and to analyze genotype by environment interactions. However, such field studies are easily affected by changes in climatic conditions at different locations and different years, and environmental conditions are difficult to control. There are many confounding factors, and repeatability is generally poor. Indoor evaluation methods have several advantages. They are not limited by seasons, they have fewer confounding factors, the environmental conditions are easily controlled, and different temperature gradients can be applied during the experiment to accurately assess the tolerance of different maize varieties to low temperature; however, one disadvantage to this method is that it is generally unable to assess genotypes. Because of environmental interaction effects, two methods of evaluation, both indoor and outdoor, were used, and an improved technical system was established for the present study. Many performance indicators have been applied to evaluate the low-temperature tolerance of maize germination, such as the germination potential, germination rate, germination index, radicle length and germ length [5, 14]. For evaluation of seed germination ability at low temperatures, the ratio (relative value) of various traits measured at low temperature and normal temperature is usually used as an index to eliminate the differences in the genetic background of different materials [12, 14].

With the above established evaluation system, a maize population panel of 222 diverse inbred lines was used for the analysis of traits correlated with low-temperature tolerance via GWAS. Candidate genes were predicted based on RNA-seq data to achieve the following objectives: (1) identify potential SNPs responsible for low-temperature tolerance during seed germination, (2) identify maize inbred lines with extremely low-temperature tolerance, and (3) predict and identify the involved candidate genes for future studies and agricultural applications.

## Results

### Low-temperature germination ability of the maize lines

**Fig. 1:**
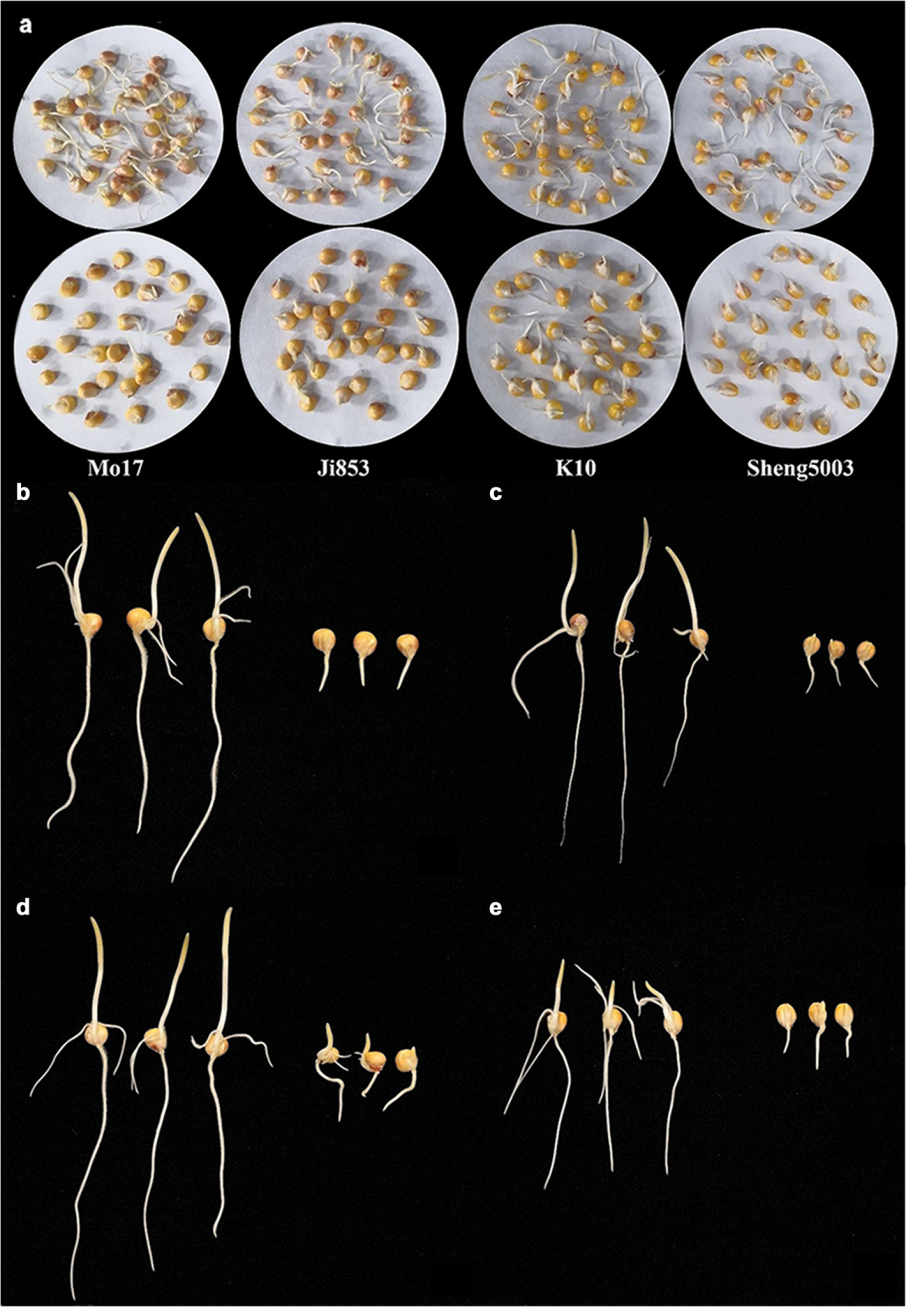
Seed germination of the four maize inbred lines under low-temperature conditions. **a** Germination rate comparison among four maize inbred lines in the low-temperature treatment (10 °C) and the control treatment (25 °C); **b** Comparison of the germ length and radicle length of maize inbred line Mo17; **c** inbred line Ji 853; **d** inbred line K10; and **e** inbred line Shen5003

The germination rate (Fig. 1a) and seedling performance (Fig. 1b, c, d, e) of the 222 maize lines were evaluated. The following 14 traits were measured: the relative germination rate (RGR), relative germ length (RGL), relative radicle length (RRL), relative radicle surface area (RRSA), relative radicle volume (RRV), relative germination index (RGI), relative vitality index (RVI), relative simple vitality index (RSVI), XiangYang relative germination rate (XYRGR), XiangYang relative germ length (XYRGL), XiangYang relative simple vitality index (XYRSVI), KeShan relative germination rate (KSRGR), KeShan relative germ length (KSRGL), and KeShan relative simple vitality index (KSRSVI). Descriptive statistics of the relative values of the germination traits under low-temperature conditions and normal conditions, including the numbers of observations (n), means, medians, standard deviations (SDs), range, kurtosis and skewness, were calculated. For most of the traits, considerable phenotypic variation was detected among the lines, with medians that ranged from 0.089 (for the RVI) to 0.788 (for the RGR). The RGR varied from 0.027 to 1.000, with a mean of 0.737, and the RVI varied from 0.001 to 0.485, with a mean of 0.101. In particular, the RGL, RRL, RRSA, and RRV of several inbred lines were larger than 1.000, suggesting that the germ and radicle growth of these inbred lines was stimulated by low-temperature treatment. In general, the segregation of most traits in the panel fit a normal distribution except for that of the RGR, RRSA, RRV, RGI, and RVI (Table 1). ANOVA revealed highly significant differences (*P* < 0.001) among the genotypes for all the relative traits. The broad-sense heritability (*H*^2^) of the 14 relative traits ranged from 87.24% (for the XYRGL) to 99.21% (for the RVI) in the association panel (Additional file 1: Table S1).

**Table 1:** Descriptive statistics for germination traits under low-temperature and normal conditions

Trait	n	Mean	Median	Range	SD	Kurtosis	Skewness
RGR	222	0.737	0.788	0.027–1.000	0.220	1.462	−1.313
RGL	222	0.486	0.434	0.087–1.229	0.220	−0.170	0.671
RRL	222	0.704	0.719	0.100–1.838	0.285	1.388	0.496
RRSA	222	0.722	0.769	0.133–2.352	0.282	5.217	0.936
RRV	222	0.739	0.737	0.173–2.650	0.325	5.171	1.244
RGI	222	0.169	0.167	0.006–0.576	0.075	4.463	1.050
RVI	222	0.101	0.089	0.001–0.485	0.067	5.511	1.694
RSVI	222	0.360	0.341	0.002–0.917	0.186	0.012	0.466
XYRGR	222	0.403	0.421	0.014–0.753	0.172	−0.633	−0.316
XYRGL	222	0.544	0.538	0.338–0.897	0.797	1.344	0.431
XYRSVI	222	0.223	0.224	0.005–0.557	0.105	−0.153	0.084
KSRGR	222	0.510	0.538	0.084–0.857	0.175	−0.399	−0.371
KSRGL	222	0.500	0.496	0.212–0.851	0.111	0.056	0.062
KSRSVI	222	0.262	0.265	0.018–0.535	0.115	−0.419	0.113

The traits were evaluated for 222 inbred lines during germination tests under normal (25 °C) and low-temperature conditions (10 °C). Fourteen relative (R) traits were derived by dividing the directly measured trait values (GR, GL, RL, RSA, RV, GI, VI SVI, XYGR, XYGL, XYSVI, KSGR, KSGL and KSVI) under low-temperature conditions by their corresponding values under normal conditions

**Fig. 2:**
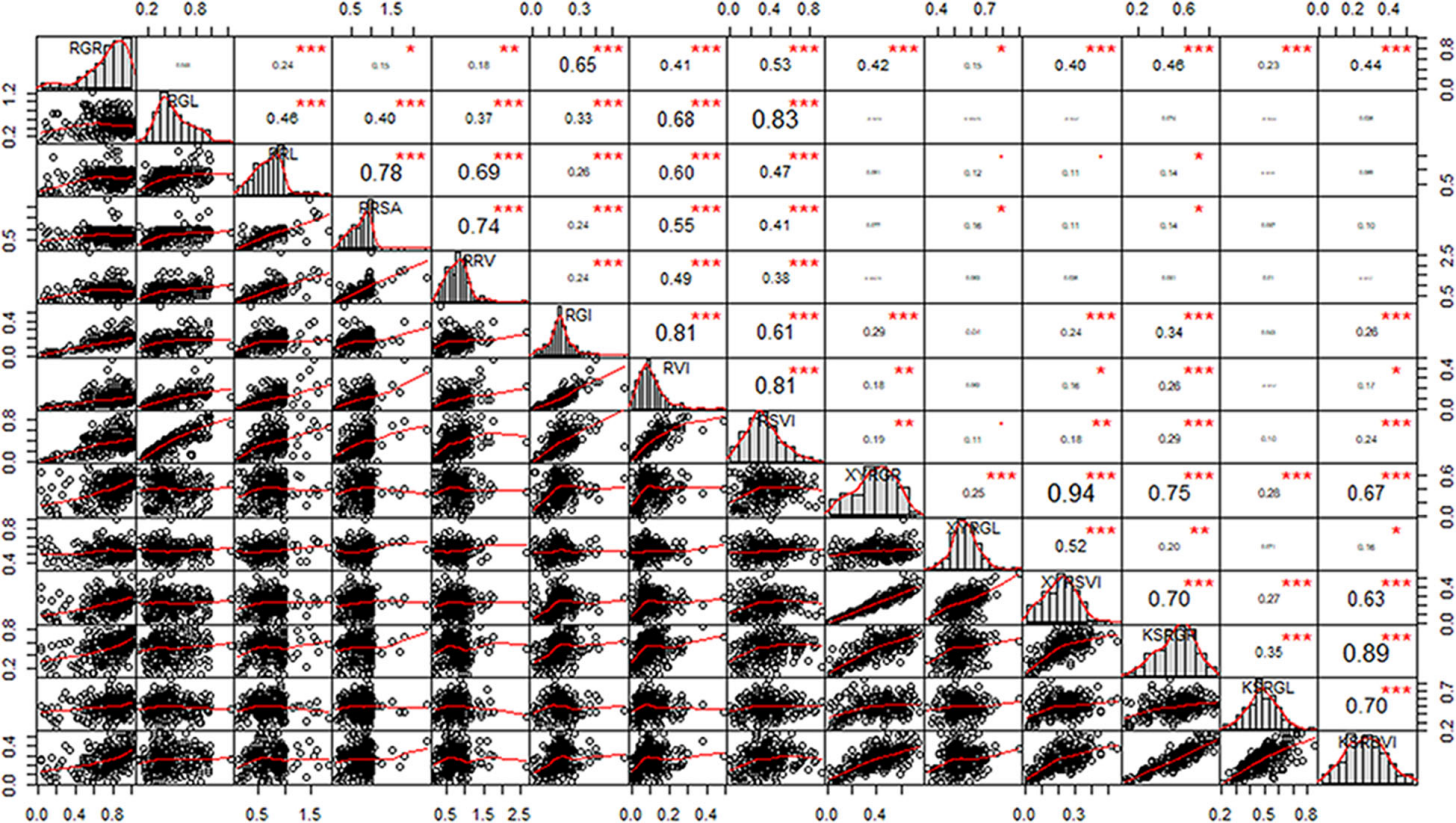
Distributions of and correlations between 14 relative phenotypic traits. The frequency distribution of each trait is shown on a central diagonal in the form of a histogram. Scatter plots of between every pair of traits are shown in the areas below the diagonal, and numerical correlation coefficients between every pair of traits are shown in the areas above in the diagonal. *, ** and *** indicate significance at *P* < 0.05, *P* < 0.01 and *P* < 0.001, respectively

In total, 14 traits, which were monomodally distributed, were measured (Fig. 2). Correlation analysis was carried out on the relative values of each index under low-temperature stress. The traits during the germination period were closely related to the regulation of maize growth (*P* < 0.05), except for the RGR and RGL. For the indoor traits, the RSVI and RGL (0.83), RVI and RGI (0.81), and RRSA and RRL (0.78) showed strong correlations (*P* < 0.001). There were significant positive correlations between the other traits, indicating that the eight traits selected could be used as important indicators for low-temperature tolerance in maize germination. Except for the XYRGL and KSRGL, most traits in the field reached a significant level of correlation (*P* < 0.05). More significant positive correlations were found between the other traits in the field, such as the XYRGR and XYRSVI (0.94) and the KSRGR and KSRSVI (0.89) (*P* < 0.001). However, correlations were weak between most traits under indoor and field conditions, such as the RSVI and XYRGL (0.11) (*P* < 0.05) (Fig. 2). Pairwise correlation coefficients among the eight indoor traits were significant and positive for the low-temperature conditions (*r* = 0.264–0.948), and pairwise correlation coefficients among the six field traits were also significant and positive for the low-temperature conditions (*r* = 0.310–0.970). Under normal conditions, most indoor traits and field traits showed a weak correlation, which may be due to the different effects of these environments (Additional file 2: Table S2).

### Population structure and LD decay

The population structure of the 222 inbred lines was calculated using STRUCTURE version 2.3 [25]. ΔK attains the peak value when the K = 4, it is indicated that the 222 inbred lines could be divided into the four subpopulations (Additional file 3: Fig. S1). A total of 40,697 high-quality SNPs were used in LD analysis for all the chromosomes. LD varied along each chromosome. When *r*^*2*^ = 0.1, the mean LD decay was 395–1125 kb across all chromosomes. The distribution of *r*^*2*^ values along with the physical distance for each chromosome is shown in Additional file 4: Table S3 and Additional file 5: Fig. S2.

### Associated SNPs for GWAS

**Fig. 3:**
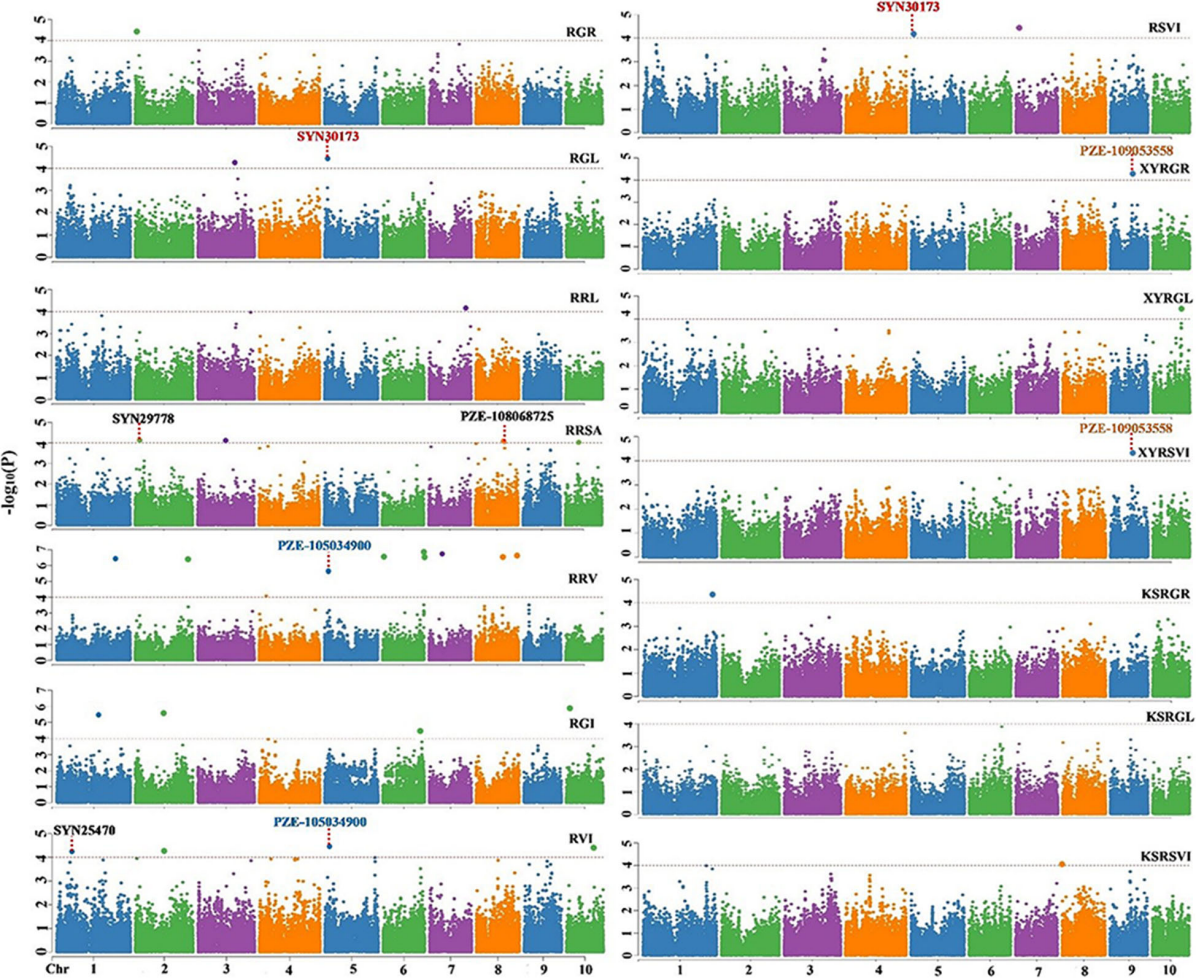
Manhattan plots of GWAS results showing the significant SNPs associated with 14 relative phenotypic traits. The traits included the following: the relative germination rate (RGR), relative germ length (RGL), relative radicle length (RRL), relative radicle surface area (RRSA), relative radicle volume (RRV), relative germination index (RGI), relative vitality index (RVI), relative simple vitality index (RSVI), XiangYang relative germination rate (XYRGR), XiangYang relative germ length (XYRGL), XiangYang relative simple vitality index (XYRSVI), KeShan relative germination rate (KSRGR), KeShan relative germ length (KSRGL), KeShan relative simple vitality index (KSRSVI)

GWAS was conducted on the 14 relative traits (RGR, RGL, RRL, RRSA, RRV, RGI, RVI, RSVI, XYRGR, XYRGL, XYRSVI, KSRGR, KSRGL, KSRSVI) for the 222 maize inbred lines. The GWAS of the SNP markers and traits was performed using the mixed-linear model (MLM) combined with population structure and kinship using TASSEL 5.0 software. A total of 30 SNPs showed a highly significant association with 13 traits except the KSRGL, which may be due to the different effects of these environments (the *P* values ranged from 1.70E-07 to 9.95E-05; Fig. 3).

Among the 30 associated SNPs, three SNPs (SYN30173, PZE-105034900, PZE-109053558) were associated with two traits (RGL and RSVI, RRV and RVI, XYRGR and XYRSVI). These SNPs were distributed on all 10 maize chromosomes, with the highest number on chromosome 2, which contained five SNPs. For each trait, such as the RGR, RRL, XYRGR, XYRGL, XYRSVI, KSRGR and KSRSVI, one SNP was found. Two SNPs were detected for the RGL, and the most significant association was for SYN30173, with a *P* value of 3.75E-05. Ten SNPs were associated with the RRV and are located on chromosomes 1 (PZE-101192647), 2 (SYN21841), 4 (PZE-104024779), 5 (PZE-105034900), and 7 (PZE-107036384). Three SNPs were located on chromosome 6 (PZE-106003222, PZE-106129965 and PZE-106130106), and two SNPs (PZE-108064544 and SYN26538) were found on chromosome 8. Four SNPs were found for the RRSA (SYN29778, PZE-108068725, PZE-103072583, PZE-110029252), RGI (PZE-101120376, PZE-102099570, PZE-106097864, SYN5516) and RVI (SYN25470, PZE-102100684, PZE-105034900, PZE-110057591). Two SNPs were associated with the RSVI—one on chromosome 5 (SYN30173) and the other on chromosome 7 (SYN4961). Thirteen relative traits contained 30 SNPs explained 12.0–28.6% of the total phenotypic variation (Table 2).

**Table 2:** SNPs for low-temperature germination-related traits detected in 222 maize inbred lines

SNP	Chromosome	Position	Bin	MAF	***P*** value	Alleles	***R***^**2**^	Trait
SYN25470	1	60,897,507	1.04	0.34	6.50E-05	A/C	0.124	RVI
PZE-101120376	1	148,125,053	1.05	0.138	3.52E-06	A/C	0.265	RGI
PZE-101192647	1	238,680,213	1.08	0.111	3.53E-07	A/G	0.286	RRV
PUT-163a-88,747,038-4526	1	282,891,355	1.11	0.279	4.32E-05	A/C	0.127	KSRGR
PUT-163a-149,007,696-748	2	8,500,779	2.02	0.077	3.60E-05	A/G	0.159	RGR
SYN29778	2	17,713,628	2.03	0.318	6.89E-05	A/C	0.151	RRSA
PZE-102099570	2	114,621,446	2.05	0.361	2.95E-06	A/G	0.207	RGI
PZE-102100684	2	117,468,629	2.05	0.267	6.27E-05	A/C	0.182	RVI
SYN21841	2	219,117,623	2.08	0.253	3.73E-07	A/C	0.272	RRV
PZE-103072583	3	115,856,771	3.04	0.346	8.88E-05	A/G	0.149	RRSA
PZE-103094415	3	151,499,271	3.05	0.182	6.34E-05	A/G	0.135	RGL
PZE-104024779	4	28,770,811	4.04	0.095	8.24E-05	A/G	0.120	RRV
SYN30173	5	10,372,835	5.02	0.401	3.75E-05/7.50E-05	A/G	0.133/0.121	RGL/RSVI
PZE-105034900	5	19,776,726	5.03	0.089	2.34E-06/3.06E-05	A/C	0.209/0.165	RRV/RVI
PZE-106003222	6	4,152,307	6.00	0.141	2.15E-07	A/G	0.259	RRV
PZE-106097864	6	151,745,619	6.05	0.137	4.23E-05	A/G	0.159	RGI
PZE-106129965	6	168,030,463	6.08	0.382	1.96E-07	A/G	0.254	RRV
PZE-106130106	6	168,266,461	6.08	0.359	2.88E-07	A/G	0.267	RRV
SYN4961	7	7,156,626	7.01	0.144	3.602E-05	A/G	0.132	RSVI
PZE-107036384	7	56,177,181	7.02	0.257	1.70E-07	A/G	0.258	RRV
PZE-107098845	7	148,186,320	7.03	0.161	7.53E-05	A/G	0.127	RRL
PUT-163a-78,076,151-4108	8	1,508,965	8.00	0.282	9.95E-05	A/C	0.142	KSRSVI
PZE-108064544	8	113,952,611	8.04	0.141	2.79E-07	A/G	0.267	RRV
PZE-108068725	8	119,362,385	8.04	0.286	9.81E-05	A/C	0.145	RRSA
SYN26538	8	169,361,414	8.08	0.224	2.24E-07	A/G	0.259	RRV
PZE-109053558	9	89,605,923	9.03	0.4	5.64E-05/5.01E-05	A/G	0.134/0.136	XYRGR/XYRSVI
SYN5516	10	8,952,194	10.02	0.301	1.21E-06	A/G	0.250	RGI
PZE-110029252	10	50,842,298	10.03	0.128	9.84E-05	A/G	0.177	RRSA
PZE-110057591	10	110,369,493	10.04	0.113	4.24E-05	A/G	0.130	RVI
PZE-110060997	10	115,266,833	10.04	0.122	4.05E-05	A/G	0.132	XYRGL

The location is indicated by the chromosome and base pair position. *P* values less than 10^−4^ corresponding to a 5% type I error are displayed in scientific notation. The frequency is indicated by the minor allele frequency (MAF). *R*^2^: the percentage of phenotypic variation

### Identification of candidate genes in maize

A total of 14 candidate genes were found to be directly associated with SNPs using the B73 RefGen_v4 Maize Gene Database (http://www.maizegdb.org/) (Table 3). Two of these genes (*Zm00001d018864* and *Zm00001d013415*) which are directly associated with SNPs, were correlated with the RSVI, while *Zm00001d013415* was also correlated with the RGL. Three candidate genes (*Zm00001d041438*, *Zm00001d010670* and*Zm00001d002676*) were associated with the RRSA, and three genes (*Zm00001d049442*, *Zm00001d010497* and *Zm00001d013794*) were associated with the RRV. Two genes (*Zm00001d029193* and *Zm00001d013794*) were associated with the RVI, while *Zm00001d013794* was correlated with both the RRV and RVI. For the RGR (*Zm00001d002243*), RGI (*Zm00001d023538*), XYRGL (*Zm00001d025380*), KSRGR (*Zm00001d034313*), and KSRSVI (*Zm00001d008224*), only one candidate gene was associated.

**Table 3:** Functions of candidate genes that are directly associated with germination traits

Trait	SNP	Gene ID	Gene function
RGR	PUT-163a-149,007,696-748	*Zm00001d002243*	uncharacterized
RGL/RSVI	SYN30173	*Zm00001d013415*	probable receptor protein kinase TMK1
RSVI	SYN4961	*Zm00001d018864*	histone deacetylase
RRSA	PZE-103072583	*Zm00001d041438*	protein ROOT HAIR DEFECTIVE 3 homolog 1
RRSA	SYN29778	*Zm00001d002676*	pseudouridine synthase family protein
RRSA	PZE-108068725	*Zm00001d010670*	glycerol-3-phosphate dehydrogenase
RRV	PZE-104024779	*Zm00001d049442*	polyadenylation and cleavage factor homolog 4
RRV	PZE-108064544	*Zm00001d010497*	uncharacterized
RRV/RVI	PZE-105034900	*Zm00001d013794*	NA
RGI	SYN5516	*Zm00001d023538*	long chain acyl-CoA synthetase 9, chloroplastic
RVI	SYN25470	*Zm00001d029193*	uncharacterized
XYRGL	PZE-110060997	*Zm00001d025380*	uncharacterized
KSRGR	PUT-163a-88,747,038-4526	*Zm00001d034313*	seed maturation protein
KSRSVI	PUT-163a-78,076,151-4108	*Zm00001d008224*	DNA-binding protein

The mean LD decay of the 10 chromosomes across the 222 maize lines was 60 kb, and the LD decay of chromosomes 1 to 10 was 55, 60, 110, 100, 60, 45, 80, 70, 60, and 110 kb (*r*^*2*^ = 0.2), respectively. A 55 kb window was selected to fall within the estimated window of LD decay on chromosome 1; 60 kb, chromosome 2; 110 kb, chromosome 3; and so on. Furthermore, an additional 68 candidate genes were identified within the LD decay regions of 26 associated SNPs (Additional file 6: Table S4).

### Differential gene expression and determination of candidate genes

**Fig. 4:**
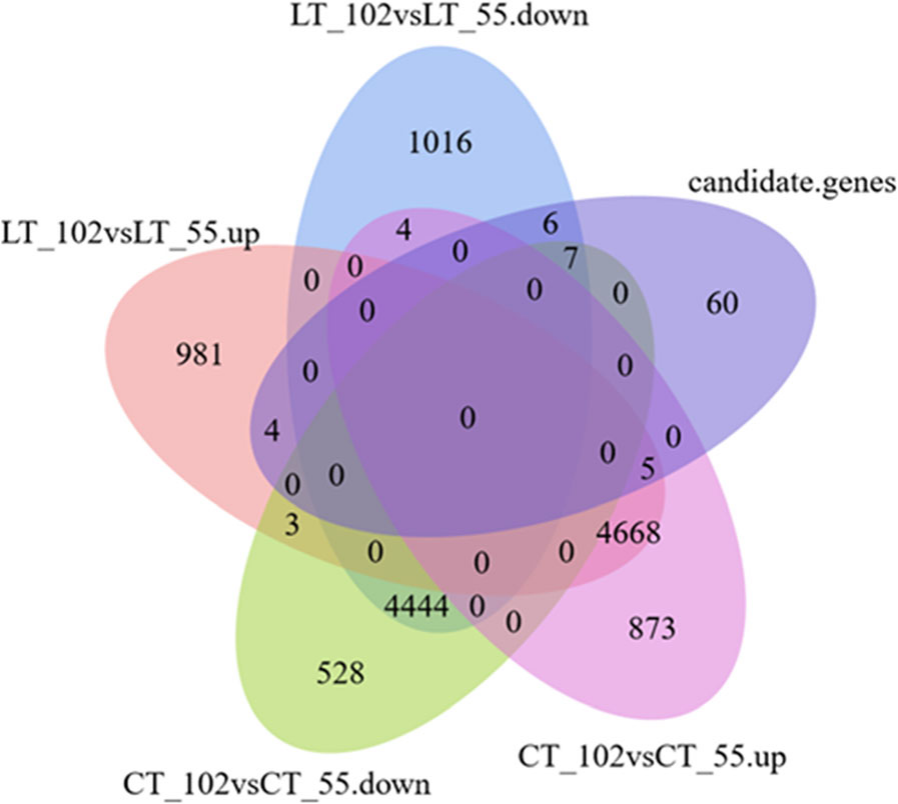
Venn diagram of DEGs distributed in maize inbred lines 102 and 55. The treatments included the normal control treatment (CT) and the low-temperature treatment (LT). The low-temperature-tolerant line (Zao 8–3) and low-temperature-sensitive line (Ji 853) are labeled “55” and “102”, respectively. The five treatment-line comparisons groups included the following: low-temperature treatment line 102 vs low-temperature treatment 55, upregulated expression (LT_102vsLT_55.up); low-temperature treatment line 102 vs low-temperature treatment 55, downregulated expression (LT_102vsLT_55.down); normal control treatment line 102 vs normal control treatment 55, upregulated expression (CT_102vsCT_55.up); normal control treatment line 102 vs normal control treatment 55, downregulated expression (CT_102vsCT_55.down); and candidate genes

To help identify candidate genes for the identified SNPs, the low-temperature-resistant maize line (Zao 8–3, referred to as 55) and the low-temperature-sensitive line (Ji 853, referred to as 102) were selected from among the 222 inbred lines, and RNA-seq analysis was performed to evaluate genome-wide gene expression levels. A total of 4982 DEGs were identified as downregulated in the CT_102vsCT_55 comparison group, 5550 DEGs were identified at upregulated in the CT_102vsCT_55 comparison group, 5477 DEGs were identified as downregulated in the LT_102vsLT_55 comparison group, and 5661 DEGs were identified as upregulated in the LT_102vsLT_55 comparison group. The results showed that 1022 DEGs were downregulated only in the LT_102vsLT_55 comparison group and that 985 DEGs were upregulated only in the LT_102vsLT_55 comparison group. Four DEGs (|log_2_FC| ≥ 0.585, *P* < 0.05) were downregulated in the LT_102vsLT_55 comparison group but upregulated in the CT_102vsCT_55 comparison group. Three DEGs (|log_2_FC | ≥ 0.585, *P* < 0.05) were upregulated in the LT_102vsLT_55 comparison groups but were downregulated in the CT_102vsCT_55 comparison group (Fig. 4).

A total of 82 candidate genes were associated with SNPs, and ten important DEGs were identified, including six candidate genes (*Zm00001d034319*, *Zm00001d025379*, *Zm00001d021653*, *Zm00001d039219*, *Zm00001d002241*, and *Zm00001d002677*) that were downregulated only in the LT_102vsLT_55 comparison group and four candidate genes (*Zm00001d002676*, *Zm00001d038373*, *Zm00001d029193*, and *Zm00001d012512*) that were upregulated only in the LT_102vsLT_55 comparison group. The putative identities of the proteins encoded by these genes are listed in Table 4. *Zm00001d034319* encodes an inositol phosphoryl ceramide-B C-26 hydroxylase, *Zm00001d025379* encodes a photoperiod responsive protein, *Zm00001d021653* encodes glucose-6-phosphate/phosphate translocator 2, *Zm00001d039219* encodes a pleckstrin homology (PH) domain-containing protein, *Zm00001d002241* encodes an AT hook motif family protein, *Zm00001d002677* encodes coiled-coil domain-containing protein 124, *Zm00001d002676* encodes a pseudouridine synthase family protein, *Zm00001d038373* encodes a fasciclin-like arabinogalactan family protein (SOS5), and *Zm00001d029193* encodes a monocopper oxidase.

**Table 4:** Candidate DEGs that are inconsistent between the CT_102vsCT_55 and LT_102vsLT_55 comparison groups

Gene ID	CT_102vsCT_55	LT_102vsLT_55	Gene function	***Arabidopsis*** best hit	Rice best hit
*Zm00001d034319*	no	down	inositol phosphoryl ceramide-B C-26 hydroxylase	(ATFAH1, FAH1) fatty acid hydroxylase 1	fatty acid hydroxylase putative expressed
*Zm00001d025379*	no	down	photoperiod responsive protein	(ATCMPG2, CMPG2) CYS MET PRO and GLY protein 2	U-box domain containing protein expressed
*Zm00001d021653*	no	down	glucose-6-phosphate/phosphate translocator 2	(ATGPT2, GPT2) glucose-6-phosphate/phosphate translocator 2	transporter family protein putative expressed
*Zm00001d039219*	no	down	uncharacterized	pleckstrin homology (PH) domain-containing protein	pleckstrin homology domain-containing protein-related taxo putative expressed
*Zm00001d002241*	no	down	uncharacterized	no	AT hook motif family protein expressed
*Zm00001d002677*	no	down	uncharacterized	no	coiled-coil domain-containing protein 124 putative expressed
*Zm00001d002676*	no	up	pseudouridine synthase family protein	pseudouridine synthase family protein	coiled-coil domain-containing protein 139 putative expressed
*Zm00001d038373*	no	up	uncharacterized	(SOS5) fasciclin-like arabinogalactan family protein	fasciclin-like arabinogalactan protein putative expressed
*Zm00001d029193*	no	up	uncharacterized	SKU5 similar 3 (sks3)	monocopper oxidase putative expressed
*Zm00001d012512*	no	up	uncharacterized	protein of unknown function (DUF3133)	expressed protein

The list consists of six parts: gene ID, gene function description, up- or downregulation in the CT_102vsCT_55 and LT_102vsLT_55 comparison group, and *Arabidopsis* and rice best hits

### Functional prediction of candidate genes

**Fig. 5:**
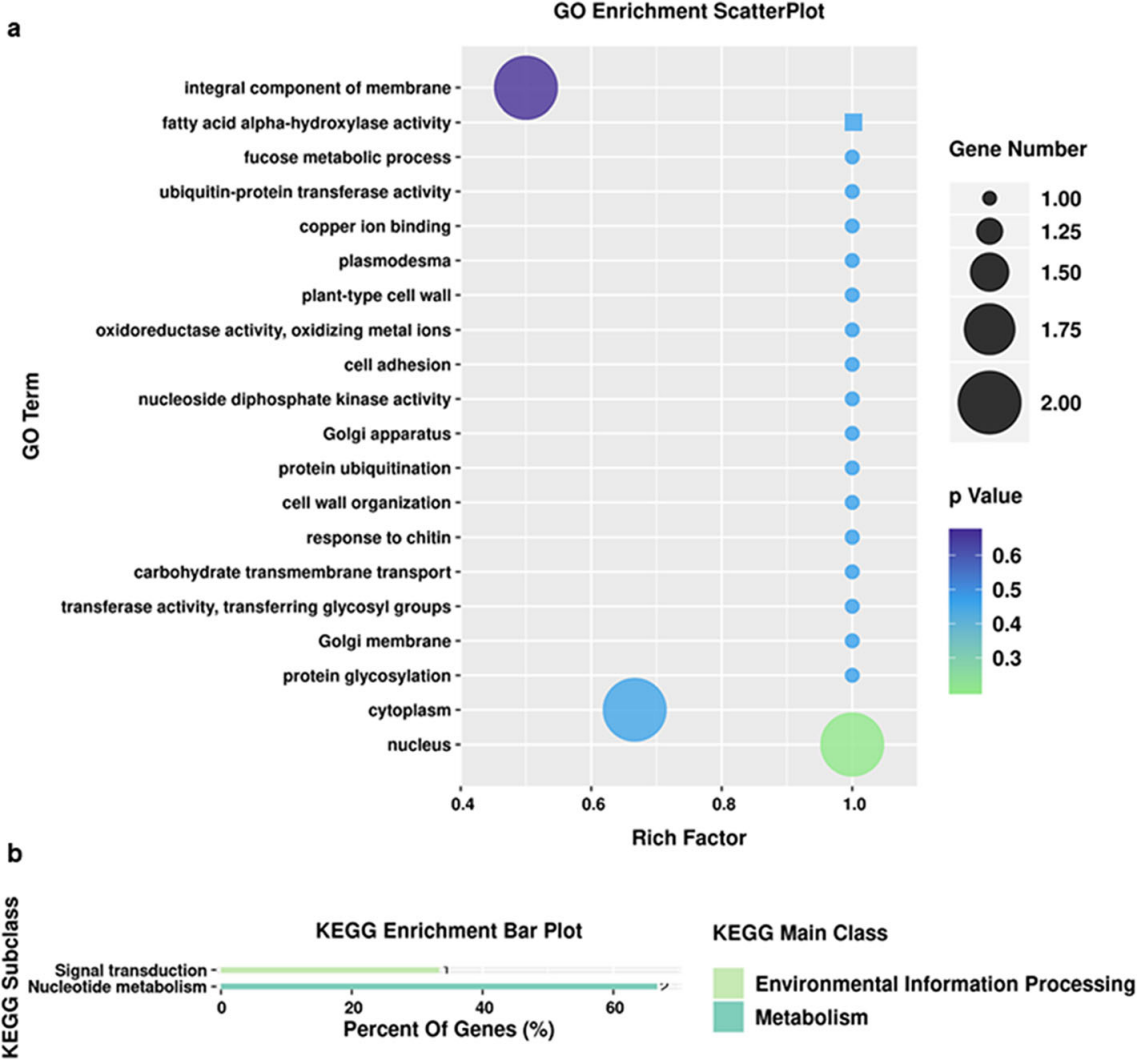
GO annotations and KEGG pathways of DEGs**. a** Bubble chart of GO classifications of the DEGs. The green circle represents the GO annotations common to the *Zm00001d039219* and *Zm00001d002676* genes*.* The purple circle represents the GO annotations common to the *Zm00001d029193* and *Zm00001d038373* genes. The blue square represents the GO annotations common to the *Zm00001d034319* gene. A total of 8 DEGs were annotated; **b** Histogram of the KEGG pathways of the annotated DEGs

A total of 26 GO terms were found to be associated with the eight candidate genes, and three KEGG pathways were found to be associated with one candidate gene (Tables 5, 6; Additional file 7: Table S5). These GO terms are associated with multiple functions. The first type of function is related to biological processes, including carbohydrate transmembrane transport (*Zm00001d021653*), fatty acid metabolic processes (*Zm00001d034319*), protein ubiquitination (*Zm00001d025379*), fucose metabolic process, protein glycosylation, and cell adhesion (*Zm00001d038373*). The second type is related to molecular functions, which involve copper ion binding and oxidoreductase activity (*Zm00001d029193*), fatty acid alpha-hydroxylase activity (*Zm00001d034319*), ubiquitin-protein transferase activity (*Zm00001d025379*), transferase activity, transferring glycosyl groups (*Zm00001d038373*), and nucleoside diphosphate kinase activity (*Zm00001d039219*). The third type of function is related to cellular components, which involve the endoplasmic reticulum membrane (*Zm00001d034319*), chloroplasts (*Zm00001d039219*), plant-type cell wall, plasmodesmata and mitochondria (*Zm00001d029193*), the endoplasmic reticulum (*Zm00001d034319*), the Golgi apparatus (*Zm00001d038373*), the cytoplasm (*Zm00001d025379* and *Zm00001d002677*), the nucleus (*Zm00001d039219* and *Zm00001d002676*), and integral components of membrane (*Zm00001d038373* and *Zm00001d029193*) (Table 5; Fig. 5a). Two KEGG pathways were identified. The first type of function is related to MAPK signal transduction, and the second type of function is related to nucleotide metabolism, including both purine metabolism and pyrimidine metabolism (Table 6; Fig. 5b).

**Table 5:** GO analysis of candidate genes related to the RNA-seq results

Gene ID	GO
*Zm00001d034319*	GO:0005783; GO:0006631; GO:0080132
*Zm00001d025379*	GO:0004842; GO:0005737; GO:0010200; GO:0016567
*Zm00001d021653*	GO:0034219; GO:0034219
*Zm00001d039219*	GO:0004550; GO:0005634; GO:0009507
*Zm00001d002241*	no
*Zm00001d002677*	GO:0003674; GO:0005737; GO:0008150
*Zm00001d002676*	GO:0005634
*Zm00001d038373*	GO:0000139; GO:0005794; GO:0006004; GO:0006486; GO:0007155; GO:0016021; GO:0016757; GO:0071555
*Zm00001d029193*	GO:0005507; GO:0009505; GO:0009506; GO:0016722; GO:0003674; GO:0005739; GO:0008150; GO:0016021
*Zm00001d012512*	no

**Table 6:** Distribution of genes and pathways related to the RNA-seq results

Pathway_ID	Pathway_name	Gene ID	KO_Entry	EC
ko00230	Purine metabolism	*Zm00001d039219*	K00940	EC:2.7.4.6
ko00240	Pyrimidine metabolism	*Zm00001d039219*	K00940	EC:2.7.4.6
ko04016	MAPK signaling pathway - plant	*Zm00001d039219*	K00940	EC:2.7.4.6

### Validation of qRT-PCR for differentially expressed genes

**Fig. 6:**
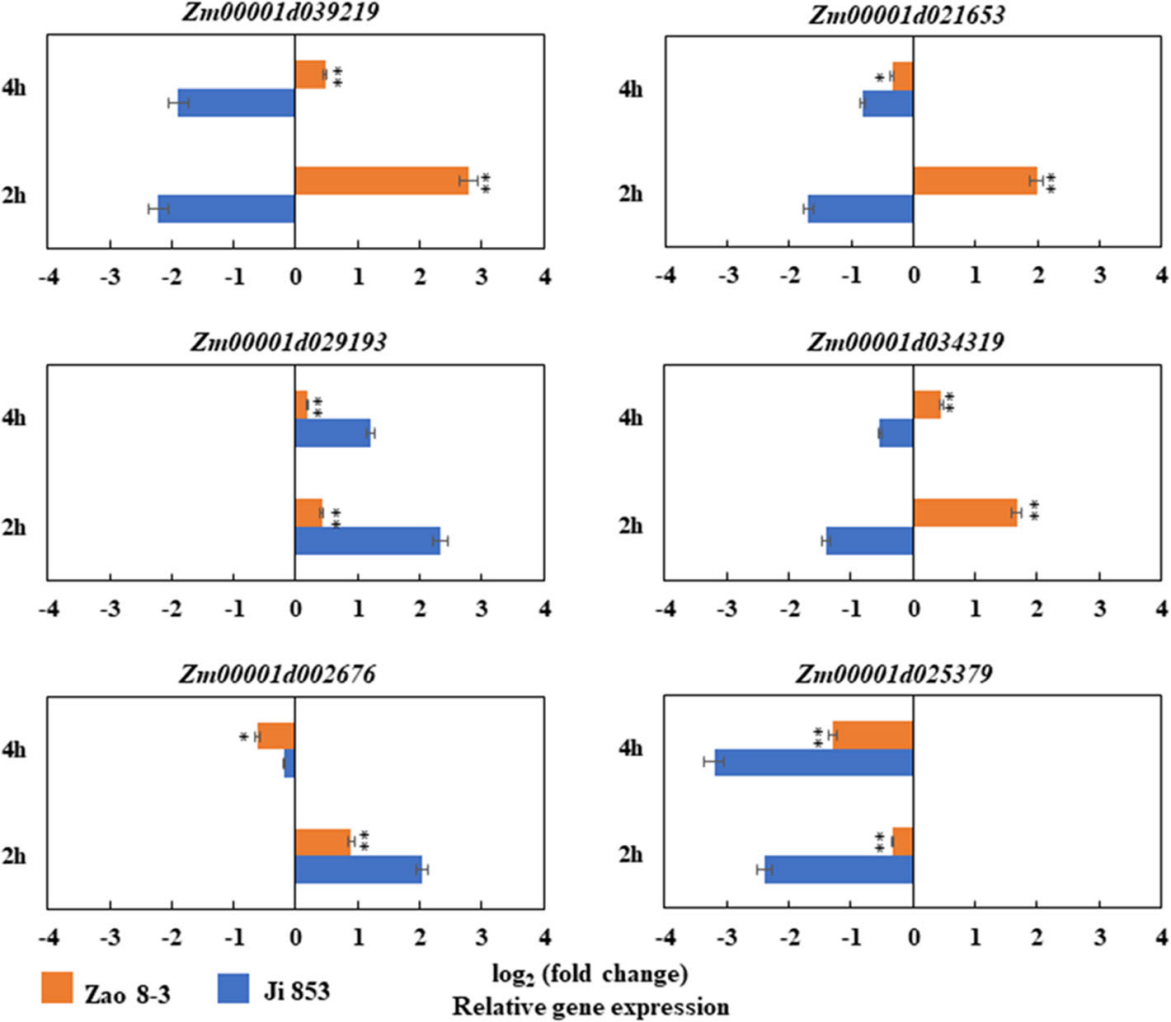
qRT-PCR validation of the GWAS and RNA-seq results. Expression of six candidate genes in the low-temperature-resistant line Zao 8–3 (referred to as 55) and the low-temperature-sensitive line Ji 853 (referred to as 102). Expression analysis was conducted on embryos that were collected at 2 h and 4 h under low (10 °C) and normal (25 °C) temperature conditions, respectively. * and ** indicate significance at *P* < 0.05 and *P* < 0.01, respectively

Six candidate genes that showed significant differential expression levels according to the RNA-seq were chosen for further qRT-PCR analysis: *Zm00001d039219*, *Zm00001d029193, Zm00001d002676*, *Zm00001d021653*, *Zm00001d034319* and *Zm00001d025379*. A comparison of the qRT-PCR results and RNA-seq data showed consistent expression trends for all six genes. Four genes (*Zm00001d039219*, *Zm00001d021653*, *Zm00001d034319*, and *Zm00001d025379*) showed significantly higher expression levels in 55 than in 102 under 2 h and 4 h of treatment with low temperatures, and two genes (*Zm00001d029193* and *Zm00001d002676*) showed significantly lower expression levels in 55 than in 102 (Fig. 6).

Two candidate genes are of particular interest: *Zm00001d039219* and *Zm00001d034319*. They showed significantly upregulated expression in 55 and showed significant downregulated expression in 102 under all conditions (both low temperatures for 2 h and 4 h) and are involved in functions related to low-temperature resistance, such as MAPK signaling and fatty acid metabolic processes.

## Discussion

### Relationship between phenotypic characteristics and low-temperature tolerance of maize

The entire growth process of maize is affected by low-temperature stress beginning at the seed germination stage. The most important indicator of low-temperature tolerance during germination is root emergence [1], which is a critical factor for plant development and yield. Low temperature stress decreases root activity, shortens the root length, and results in fewer lateral roots in affected plants [26]. In accordance with previous studies, root and shoot growth were evaluated in controlled growth chambers under a range of temperature regimes [12]. Low-temperature stress also affects maize seedlings after they germinate. It was found that the relative water content, leaf area and leaf dry weight, plant height, root length, stem length and dry weight, and whole-plant fresh weight can be affected [12, 13, 27]. In our study, low-temperature conditions were defined as 10 °C and, for normal conditions, 25 °C. The germination traits assessed were radicle length, radicle surface area, and radicle volume. We observed a large phenotypic variation in radicle length among the 222 maize inbred lines under low-temperature conditions and a strong correlation between radicle length and germination rate. However, their genetic loci were different, indicating that different mechanisms are involved. Our study focused on germination, which was defined as radicle emergence from seeds, under low-temperature conditions. The 30 associated SNPs and two candidate genes identified in this study provide valuable resources for future studies to improve the understanding of the genetic underpinnings of low-temperature tolerance in maize and to improve maize varieties through breeding.

### Integrating GWAS data and RNA-seq data for candidate gene prediction

GWAS has been widely used to identify potential candidate genes for important abiotic stress traits in maize [28], but there are still the problems such as the false positives and so on. RNA-seq has become the preferred technique for detecting genome-wide gene expression patterns [29]. However, it is difficult to identify potential key candidate genes as large amounts of DEG are usually obtained through RNA-seq. Recently, the method of integrating GWAS and RNA-seq has been widely adopted to predict candidate genes. For example, a method combining GWAS results with linked DEGs and co-expression network analysis was used to identify seven candidate genes that respond to drought stress in maize [28]. We identified 10 candidate genes related to seed germination at low temperatures through this method. Among them, the two candidate genes, *Zm00001d039219* and *Zm00001d034319* could provide valuable information for understanding the genetic mechanism of low-temperature tolerance during maize germination.

### Consistent SNPs in previous reports

To evaluate the reliability of SNPs detected in this work, we compared the 30 SNPs identified in the present study to those identified in several related publications. Three SNPs overlapped with the physical positions of published QTLs (Additional file 8: Table S6). QTL-8 for ФPSII at the seedling stage [30] was consistent with PUT-163a-149,007,696-748. Two SNPs (PZE-102099570 and PZE-102100684) on chromosome 2 were located in QTL regions associated with straw dry weight [31]; leaf greenness (SPAD) and trapping efficiency of PSII (*F′v*/*F′m*)at 15 °C [8]. In addition, a candidate gene (*Zm00001d010671*) that was strongly correlated with a nearby SNP (PZE-108068725) was supported by a previously identified candidate gene [5]. Thus, our analysis successfully revealed the SNPs known to be associated with low-temperature tolerance, indicating that the identified SNPs from the present study are highly reliable for use in gene cloning and maize breeding.

### MAPK signaling pathways in response to low-temperature stress

MAPKs are serine–threonine kinases that mediate intracellular signaling and play vital roles in regulating plant growth, development, and stress responses. At present, many protein kinase genes, including those encoding MAPKs, have been shown to mediate the transduction of abiotic stress response signals [32]. In plants, the accumulation of permeants and antioxidants can be induced by low temperature, drought and salt stress, which is mediated by MAPK pathways in yeast and animals [33]. These MAPK pathways are activated by different stimuli via receptors such as protein tyrosine kinases, G-protein-coupled receptors and two-component histidine kinases. *Arabidopsis* has approximately 60 MAPKKKs, 10 MAPKKs, and 20 MAPKs. These kinases can be activated by low temperature and other abiotic stresses and are thought to be important components of abiotic stress signaling [34]. In *Medicago sativa*, low-temperature treatment results in the activation of a MAPK within ten min [35]. Similarly, in tobacco cells, a MAPK and another protein kinase were shown to be activated by osmotic stress in response to Ca^2+^ or in an ABA-independent manner [36]. The maize gene *ZmMAPK5* showed increased expression in response to specific low-temperature treatments [22]. To date, different mechanisms underlying the low-temperature response have been proposed, and relevant coordinated regulatory networks have been analyzed in rice and *Arabidopsis*. In particular, the signal transduction pathways between MPK activation and ICE1 stability at low temperatures have been elucidated. This marks an important breakthrough in the field of plant regulation in response to low temperature [37–39] and reveals the important role of MAPK cascade signals. A candidate gene (*Zm00001d039219*) reported in this study is related to the MAPK signaling pathway, which may be related to low-temperature tolerance in maize.

### Functional analysis of candidate genes

Two candidate genes (*Zm00001d039219* and *Zm00001d034319*) have putative functions related to low-temperature resistance, such as MAPK signaling [22] and fatty acid hydroxylase activity [40], in maize and other species. *Zm00001d039219* encodes a pleckstrin homology (PH) domain-containing protein, which is homologous to *Arabidopsis AT4G23895* and rice *LOC_Os05g51710.1*. Pleckstrin homology (PH) domains are typically involved in targeting proteins to the appropriate cellular location and in protein-protein interactions. Despite minimal sequence conservation, they share a common electrostatically polarized fold. Some (< 10%) PH domains bind phosphoinositide phosphates (PIPs) with high specificity and affinity. They are found in a wide range of cellular signaling proteins, including serine/threonine kinases, adaptors, cytoskeletal-associated molecules, lipid-associated enzymes, tyrosine kinases, regulators of G-proteins, and endocytotic GTPases [41]. The putative protein encoded by *Zm00001d034319* is an inositol phosphorylceramide-B C-26 hydroxylase that belongs to the fatty acid hydroxylase superfamily and shares sequence homology with the fatty acid hydroxylase in *Arabidopsis* (*AT2G34770*) and in rice (*LOC_Os03g56820.1*). The fatty acid hydroxylase superfamily includes both fatty acid and carotene hydroxylases and sterol desaturases. Beta-carotene hydroxylase hydroxylates beta-carotene in zeaxanthin synthesis and may be involved in other pathways. Other family members include C-5 sterol desaturases and C-4 sterol methyl oxidases. The family members containing two copies of the HXHH motif are involved in cholesterol biosynthesis and biosynthesis of plant cuticular wax; these members are typically integral membrane proteins [41]. Maize cell membrane fluidity significantly decreases under low-temperature stress, and the normal physiological function of membrane-bound proteins is lost. Alteration of the composition of membrane lipid fatty acids through genetic manipulation was shown to improve the low-temperature tolerance of plants [40].

In conclusion, two candidate genes (*Zm00001d039219* and *Zm00001d034319*) were found to have significantly different gene expression levels under low-temperature treatment in resistant and sensitive maize lines. Homologs of these genes in *Arabidopsis* and rice have functions related to low-temperature resistance to stress, so these genes are attractive candidate genes for involvement in low-temperature tolerance in maize.

## Conclusion

In the present study, we contributed to the understanding of the genetic control of low-temperature tolerance in maize at the germination stage by the use of a panel of 222 maize inbred lines. Through GWAS, RNA-seq analysis, and validation via qRT-PCR, the two candidate genes (*Zm00001d039219* and *Zm00001d034319*) were found to have significantly different gene expression levels under low-temperature treatment in resistant and sensitive maize lines.

## Methods

### Plant materials

A panel consisting of 222 maize inbred lines was used for the association study (This seeds were originally acquired from researcher Xinhai Li and associate researcher Jianfeng Weng, Institute of Crop Science, Chinese Academy of Agricultural Sciences) [25, 42, 43]. These inbred lines were shown to have a wide range of variation in yield components and biotic stress tolerance. They are generally grown in the Yellow and Huai River valley regions, which are in northeastern and southwestern China [42]. Seeds were produced in the spring of 2016 by manual self-pollination of each plant in at Harbin city, Heilongjiang Province, under temperate climatic conditions. Plants were well managed and were kept free of any disease, insect or weed issues during the whole growing season. Harvested seeds were fully dried and then stored at 4 °C. The 222 maize inbred lines used for the GWAS had germination rates greater than 90% at 25 °C in previous studies in our laboratory (Additional file 9: Table S7).

Two maize lines that were resistant (Zao 8–3, referred to as 55) and sensitive (Ji 853, referred to as 102) to low temperature were selected from among the 222 inbred lines, and RNA-seq was performed to evaluate whole-genome gene expression levels.

### Seed germination and field experiments

The surface of the seeds was disinfected with 1% sodium hypochlorite (NaOCl) for 5 min, and the seeds were rinsed three times with sterile distilled water for germination experiments. After 6 h of imbibition at normal temperature (25 °C), in accordance with the International Seed Testing Association (https://www.seedtest.org/en/home.html, ISTA) protocol, germination experiments were performed on germination paper in a dark chamber at 10 °C for low-temperature conditions (treatment) and at 25 °C for normal conditions (control). The germination paper was wetted, and 50 sterilized seeds were sown on one piece, which was then covered by another piece of wet paper towel [5]. Seed germination was defined as an observed radicle emergence of 0.5 cm. After a treatment incubation at 10 °C for 31 days and recovery at 15 °C for 7 days or a control incubation at 25 °C for 6 days, radicle traits were measured by an Epson Perfection V800 scanner, and Regent WinRHIZO (Canada) software was used for the data analysis. For each condition (treatment and control), three independent experiments were conducted per line.

The experimental field sites were located in XiangYang, Harbin and KeShan of Qiqihar in 2018. The seeds of the low-temperature treatment group were sown when the ground temperature was consistently approximately 6 °C. The normal sowing time was used as a control. The experiment involved a random block design with three replications, and the seed germination number and seedling length were determined.

### Collection of phenotypic data

The germination rate (GR) was expressed as the percentage of germinating plants out of the total number of seeds used. The germ traits and radicle traits were measured using an Epson Perfection V800 scanner. The germ trait was the germ length (GL), and the radicle traits included the radicle length (RL), radicle surface area (RAS) and radicle volume (RV). All traits were measured as the mean of 10 seedlings. The germination index of plants was calculated from 19 to 31 days at 3-day intervals at 10 °C and was calculated daily, from 3 to 6 days at 25 °C. The calculated traits were included the germination index (GI), vitality index (VI) and simple vitality index (SVI), as follows:

$$ \mathrm{GI}=\sum \frac{\mathrm{Gt}}{\mathrm{Dt}} $$

where Gt is the number of germinating plants on a given day (Dt, days after sowing). VI = TL × GI, where TL is the total length of the seedling (including both the germ length and radicle length) on last day and GI is the germination index. SVI = GL × GR, where GR is the germination rate and GL is the germ length.

The field experiment statistics involving the germination rate were calculated on the last day of the XiangYang (XYGR) and KeShan (KSGR) tests, the germ length for XiangYang (XYGL) and KeShan (KSGL) was measured by a ruler, and a simple vitality index was calculated for both locations (XYRSVI and KSRSVI). Each experimental replicate consisted of 50 seedlings, and the mean of 10 seedlings was used for the XYGL and KSGL.

The relative performance of the 14 traits (RGR, RGL, RRL, RRSA, RRV, RGI, RVI, RSVI, XYRGR, XYRGL, XYRSVI, KSRGR, KSRGL, and KSRSVI) was calculated simply as the ratios of the mean values of measurements (*n* = 3) taken under low-temperature stress conditions and control conditions, and these ratios were used as indicators for low-temperature tolerance.

IBM SPSS Statistics version 20.0 software (https://www.ibm.com/support/pages/node/230551) was used to analyze the phenotypic data, including the range, mean, median, standard deviation, kurtosis and skewness of each relative trait. Phenotypic correlations were analyzed using IBM SPSS Statistics version 20.0 software and with the ‘PerformanceAnalytics’ package in R software. ANOVA for the 14 relative traits was performed for each association panel using the ‘lme4’ function in the R package ‘lme4’. The broad-sense heritability (*H*^2^) at each location was estimated using the following formula: *H*^2^ = δ^2^g/(δ^2^g + δ^2^/r). The *H*_b_^2^ in cross-locations was estimated using the following formula: ${H}_b^2={\updelta}^2\mathrm{g}/\left({\updelta}^2\mathrm{g}+{\updelta}^2\mathrm{g}\mathrm{e}/\mathrm{n}+{\updelta}^2/\mathrm{n}\mathrm{r}\right)$. δ^2^g, δ^2^ge, and δ^2^ are estimates of the genetic, G × E and error variances, and n and r are the number of environments and replications per environment, respectively [44].

### Genotypic data and GWAS

Genotyping was carried out on the association panel using an Illumina Maize SNP50 BeadChip, which revealed 56,110 SNPs in the population and was filtered such that SNPs with a missing percentage > 20%, SNPs with a minor allele frequency (MAF) < 0.05, and SNPs with a heterozygosity > 20% were removed [25, 42, 43]. In total, 40,697 SNPs were used for the association analysis, with a MAF of > 0.05 in the population. Using the STRUCTURE 2.3 software, 7742 distributed SNP datasets were assessed for structural parameters [45]. ∆K was calculated using StructureHarvester [46]. The kinship information for 222 inbred lines was estimated using the software TASSEL 5.0.

The GWAS was performed in accordance with the MLM in TASSEL 5.0 [47], and the following 14 traits were used for association analysis: the RGR, RGL, RRL, RRSA, RRV, RGI, RVI, RSVI, XYRGR, XYRGL, XYRSVI, KSRGR, KSRGL and KSRSVI. GEC software (http://grass.cgs.hku.hk/gec/estimateB.php?function=Bonferroni) was used to calculate the effective number of markers (Ne) and to calculate the recommended threshold (0.05/Ne) as the basis for whether the 14 trait values were significantly correlated with a given SNP. Because a Bonferroni correction (0.05 / 23,398 = 2.140e-6) was too conservative (there were very few SNPs significantly associated with the 14 traits), a less stringent threshold of -log_10_(*P*) > 4 was used to detect significant association signals [25, 43, 48]. Manhattan plots were subsequently generated by the CMplot package in R software.

The linkage disequilibrium measurement parameter *r*^*2*^ was used to estimate the LD between all SNPs with less than 25% missing data for each chromosome via the software PopLDdecay 3.30 (https://github.com/BGI-shenzhen/PopLDdecay). All significantly associated SNPs on the same chromosome whose physical distance was less than the LD decay distance were defined as one site, and the range of each LD decay upstream and downstream of the SNP of the -log_10_(*P*) peak for each site was used to mine candidate genes. The B73 RefGen_V4 gene model from the maizeGDB website (http://www.maizegdb.org/) was used to map the loci and to retrieve genetic information.

### RNA-seq and analysis of DEGs

The low-temperature-resistant line Zao 8–3 (referred to as 55) and a low-temperature-sensitive line Ji 853 (referred to as 102) were used in this study. Before imbibition, the seeds were sterilized and rinsed as described above. The seeds imbibed at normal temperature (25 °C) for 6 h and then were sampled at 2 h after imbibition on the germination paper under the ‘normal’ condition (25 °C) for CK_102 and CK_55 and under low-temperature conditions (10 °C) for LT_102 and LT_55. Embryos were isolated from the seeds of each sample, frozen in liquid nitrogen, and stored at − 80 °C for RNA extraction. There were three biological replicates for each treatment. The maize RNA samples were sent to LC-BIO (Hangzhou, China) for library construction and sequencing. Briefly, for each total RNA sample, the genomic DNA was removed by DNase I treatment, after which the mRNA was enriched using oligo (dT) magnetic beads and subsequently fragmented. Random hexamer primers were used for the synthesis of double-strand cDNA, which was further modified by end repair and 3′-polyadenylation. The fragments were amplified for sequencing via an Illumina NovaSeq 6000. The maize genome database ZmB73RefGenv4 (ftp://ftp.ncbi.nlm.nih.gov/genomes/all/GCF/000/005/005/GCF_000005005.2_B73_RefGen_v4) was used as the reference genome. The read count for each gene was obtained from the mapping results and normalized to fragments per kilobase of transcript per million mapped reads (FPKM). The default threshold for a significant difference in gene expression was set to |log_2_FC| ≥ 0.585 and *p* < 0.05. The raw sequence data are available in the NCBI GEO database under accession number GSE146666 (GSM4403922- GSM4403933).

### Identification and annotation of candidate genes

GO enrichment analysis was used to identify all the GO terms of the genes whose expression was significantly enriched compared to the background from among the lists of DEGs and to filter the DEGs corresponding to specific biological functions. All DEGs were categorized and grouped based on their GO terms using the publicly available database at http://www.geneontology.org/. The gene numbers were calculated for every term. A hypergeometric test and GO Term Finder (http://www.yeastgenome.org/help/analyze/go-term-finder) were used to identify significantly enriched GO terms. The three categories, ‘biological process’, ‘cellular component’, and ‘molecular function’, were overrepresented and were further filtered using the Bonferroni multitest adjustment method and Fisher’s exact test [49, 50]. To further understand the biological functions of the DEGs, the significantly enriched metabolic or signal transduction pathways were identified using KEGG pathway enrichment analysis [51] (http://www.genome.ad.jp/kegg/kegg2.html). Genes were annotated using the maize GDB (http://www.maizegdb.org) and NCBI databases (http://www.ncbi.nlm.nih.gov/).

### qRT-PCR-based validation of candidate genes

The total RNA samples used for transcriptome sequencing of the low-temperature-resistant line Zao 8–3 (referred to as 55) and the low-temperature-sensitive line Ji 853 (referred to as 102) were also subjected to qRT-PCR. For each sample, 500 ng of RNA was used for reverse transcription with a *TransScript*® One-Step gDNA Removal Kit in conjunction with cDNA Synthesis SuperMix (TransGen Biotech, Beijing, China). qRT-PCR was carried out using an Analytik Jena Real Time PCR Detection System, with the maize actin gene serving as a control. Gene-specific primers were designed using Primer 5.0 software (Additional file 10: Table S8). The 20 μl reaction mixtures consisted of 2 μl of diluted cDNA, 0.4 μl of reverse and forward primers, 7.2 μl of ddH_2_O and 10 μl of 2× *TransStart*® Tip Green qPCR SuperMix (TransGen Biotech, Beijing, China). The gene expression level was calculated using the relative 2^-∆∆CT analytical method [52]. For each sample, three biological replicates were included, with each biological replicate consisting of three technical repeats. For gene expression, the mean value of three replicates was used.

## Supplementary information

**Additional file 1 Table S1.** ANOVA results and heritability of 14 traits of the lines composing the association panel.

**Additional file 2 Table S2.** Correlation coefficients (r) between 14 phenotypic traits of 222 maize inbred lines under normal and low-temperature conditions.

**Additional file 3 Figure S1.** Estimated population structure of 222 maize inbred lines.

**Additional file 4 Table S3.** Mean of LD decay distance of the 10 chromosomes for *r*^*2*^ values which are equal to greater than 0.1 and 0.2.

**Additional file 5 Figure S2.** Illustration of whole-genome LD in a panel of 222 maize lines.

**Additional file 6 Table S4.** Functions of an additional 68 candidate genes identified within a LD decay of 26 associated SNPs.

**Additional file 7 Table S5.** Twenty-six GO terms associated with eight candidate genes.

**Additional file 8 Table S6.** SNPs or candidate genes that overlap with published QTLs.

**Additional file 9 Table S7.** Germination rates of 222 maize inbred lines at 25 °C.

**Additional file 10 Table S8.** Primer sequences used for quantitative real-time PCR.

## Data Availability

The raw RNA-seq data are available in the NCBI GEO database under accession number GSE146666 (https://www.ncbi.nlm.nih.gov/geo/query/acc.cgi?acc=GSE146666).

## Abbreviations

GWAS: Genome-wide association study
SNP(s): Single nucleotide polymorphism(s)
RNA-seq: RNA-sequencing
LD: Linkage disequilibrium
DEGs: Differentially expressed genes
FC: Fold change
GO: Gene ontology
KEGG: Kyoto encyclopedia of genes and genomes
qRT-PCR: Quantitative real-time PCR
RGR: Relative germination rate
RGL: Relative germ length
RRL: Relative radicle length
RRSA: Relative radicle surface area
RRV: Relative radicle volume
RGI: Relative germination index
RVI: Relative vitality index
RSVI: Relative simple vitality index
XYRGR: XiangYang relative germination rate
XYRGL: XiangYang relative germ length
XYRSVI: XiangYang relative simple vitality index
KSRGR: KeShan relative germination rate
KSRGL: KeShan relative germ length
KSRSVI: KeShan relative simple vitality index
MLM: Mixed-linear model
FPKM: Fragments per kilobase of transcript per million mapped reads
SD: Standard deviation
MAPK: Mitogen-activated protein kinase
PH: Pleckstrin homology
PIPs: Phosphoinositide phosphates
PM: Plasma membrane
