# Supplementary material for: Identification of candidate tolerance genes to low-temperature during maize germination by GWAS and RNA-seqapproaches
Source: BMC Plant Biol. 2020 Jul 14;20:333. doi: 10.1186/s12870-020-02543-9 (PMC7362524; doi:10.1186/s12870-020-02543-9)
Supplement: Supplementary file 1 — Additional file 1 Table S1. ANOVA results and heritability of 14 traits of the lines composing the association panel. [file 12870_2020_2543_MOESM1_ESM.docx]

**Additional file 1:**

**Table S1** ANOVA results and heritability of 14 traits of the lines composing the association panel

| **Traits** | **Env** | **Genotype**  **variance** | **Environment**  **variance** | **Error variance** | **Gen×Env**  **variance** | ***H*^2^ (%)** |
| --- | --- | --- | --- | --- | --- | --- |
| RGR | E1 | 0.0474*** |  | 0.0034*** |  | 97.64 |
| RGL | E1 | 0.0474 *** |  | 0.0031*** |  | 98.40 |
| RRL | E1 | 0.0787*** |  | 0.0090*** |  | 97.21 |
| RRSA | E1 | 0.0775*** |  | 0.0086*** |  | 97.31 |
| RRV | E1 | 0.1023*** |  | 0.0120*** |  | 97.16 |
| RGI | E1 | 0.0055*** |  | 0.0002* |  | 98.68 |
| RVI | E1 | 0.0045*** |  | 0.0001 |  | 99.21 |
| RSVI | E1 | 0.03401*** |  | 0.0011*** |  | 98.90 |
| XYRGR | E2 | 0.0269*** |  | 0.0080*** |  | 91.03 |
| XYRGL | E2 | 0.0055*** |  | 0.0024*** |  | 87.24 |
| XYRSVI | E2 | 0.0101*** |  | 0.0028*** |  | 91.45 |
| KSRGR | E3 | 0.0281*** |  | 0.0075*** |  | 91.78 |
| KSRGL | E3 | 0.0110*** |  | 0.0038*** |  | 89.78 |
| KSRSVI | E3 | 0.0120*** |  | 0.0032*** |  | 91.89 |
| RGR | E1/E2/E3 | 0.0187* | 0.0290* | 0.0063* | 0.0154* | 76.23 |
| RGL | E1/E2/E3 | 0.0000 | 0.0013** | 0.0028** | 0.0204** | 0.57 |
| RSVI | E1/E2/E3 | 0.0054* | 0.0050* | 0.0024* | 0.0133* | 53.38 |
| RGR | E2/E3 | 0.0226 | 0.0057 | 0.0078 | 0.0049 | 85.74 |
| RGL | E2/E3 | 0.0006* | 0.0009* | 0.0031* | 0.0076* | 12.70 |
| RSVI | E2/E3 | 0.0075* | 0.0008* | 0.0030* | 0.0035* | 76.80 |

“Env”: the specific environment: E1 is indoor; E2 is 2017 XiangYang; E3 is 2017 KeShan, “Gen × Env”: Genotype×Environment. *H*^2^: represented broad-sense heritability. *, ** and *** represent indicate significant level at *P* < 0.05, *P* < 0.01 and *P* < 0.001, respectively.
