# Supplementary material for: Identification of candidate tolerance genes to low-temperature during maize germination by GWAS and RNA-seqapproaches
Source: BMC Plant Biol. 2020 Jul 14;20:333. doi: 10.1186/s12870-020-02543-9 (PMC7362524; doi:10.1186/s12870-020-02543-9)
Supplement: Supplementary file 2 — Additional file 2 Table S2. Correlation coefficients (r) between 14 phenotypic traits of 222 maize inbred lines under normal and low-temperature conditions. [file 12870_2020_2543_MOESM2_ESM.docx]

**Additional file 2:**

**Table S2** Correlation coefﬁcients (r) between 14 phenotypic traits of 222 maize inbred lines under normal and low-temperature conditions

| **Env.** | **Trait** | **GR** | **GL** | **RL** | **RSA** | **RV** | **GI** | **VI** | **SVI** | **XYGR** | **XYGL** | **XYSVI** | **KSGR** | **KSGL** | **KSSVI** |
| --- | --- | --- | --- | --- | --- | --- | --- | --- | --- | --- | --- | --- | --- | --- | --- |
| Low tempera-ture | GR | 1 |  |  |  |  |  |  |  |  |  |  |  |  |  |
|  | GL | 0.272** | 1 |  |  |  |  |  |  |  |  |  |  |  |  |
|  | RL | 0.330** | 0.649** | 1 |  |  |  |  |  |  |  |  |  |  |  |
|  | RSA | 0.293** | 0.568** | 0.810** | 1 |  |  |  |  |  |  |  |  |  |  |
|  | RV | 0.272** | 0.478** | 0.674** | 0.834** | 1 |  |  |  |  |  |  |  |  |  |
|  | GI | 0.948** | 0.316** | 0.331** | 0.287** | 0.264** | 1 |  |  |  |  |  |  |  |  |
|  | VI | 0.705** | 0.733** | 0.779** | 0.667** | 0.565** | 0.762** | 1 |  |  |  |  |  |  |  |
|  | SVI | 0.687** | 0.847** | 0.618** | 0.552** | 0.472** | 0.704** | 0.922** | 1 |  |  |  |  |  |  |
|  | XYGR | 0.433** | 0.167* | 0.260** | 0.199** | 0.117 | 0.445** | 0.401** | 0.351** | 1 |  |  |  |  |  |
|  | XYGL | 0.225** | 0.119 | 0.195** | 0.159* | 0.100 | 0.258** | 0.269** | 0.213** | 0.458** | 1 |  |  |  |  |
|  | XYSVI | 0.415** | 0.166** | 0.258** | 0.199** | 0.114 | 0.432** | 0.393** | 0.340** | 0.970** | 0.633** | 1 |  |  |  |
|  | KSGR | 0.485** | 0.281** | 0.279** | 0.234** | 0.134* | 0.506** | 0.469** | 0.450** | 0.705** | 0.391** | 0.687** | 1 |  |  |
|  | KSGL | 0.255** | 0.161* | 0.139* | 0.142* | 0.117 | 0.286** | 0.232** | 0.241** | 0.365** | 0.310** | 0.361** | 0.448** | 1 |  |
|  | KSSVI | 0.451** | 0.271** | 0.253** | 0.226** | 0.137* | 0.474** | 0.431** | 0.425** | 0.656** | 0.387** | 0.641** | 0.922** | 0.719** | 1 |
| Normal  control | GR | 1 |  |  |  |  |  |  |  |  |  |  |  |  |  |
|  | GL | 0.141* | 1 |  |  |  |  |  |  |  |  |  |  |  |  |
|  | RL | 0.120 | 0.396** | 1 |  |  |  |  |  |  |  |  |  |  |  |
|  | RSA | 0.176** | 0.301** | 0.784** | 1 |  |  |  |  |  |  |  |  |  |  |
|  | RV | 0.230** | 0.284** | 0.591** | 0.805** | 1 |  |  |  |  |  |  |  |  |  |
|  | GI | 0.336** | 0.521** | 0.072 | 0.103 | 0.177** | 1 |  |  |  |  |  |  |  |  |
|  | VI | 0.300** | 0.853** | 0.463** | 0.396** | 0.387** | 0.825** | 1 |  |  |  |  |  |  |  |
|  | SVI | 0.233** | 0.995** | 0.400** | 0.312** | 0.302** | 0.542** | 0.867** | 1 |  |  |  |  |  |  |
|  | XYGR | -0.009 | -0.007 | -0.041 | -0.066 | -0.072 | 0.156* | 0.080 | -0.010 | 1 |  |  |  |  |  |
|  | XYGL | 0.097 | 0.049 | 0.123 | 0.036 | 0.010 | -0.009 | 0.047 | 0.057 | 0.119 | 1 |  |  |  |  |
|  | XYSVI | 0.058 | 0.017 | 0.040 | -0.024 | -0.043 | 0.112 | 0.084 | 0.021 | 0.806** | 0.674** | 1 |  |  |  |
|  | KSGR | 0.096 | 0.152* | 0.113 | 0.049 | -0.030 | 0.268** | 0.252** | 0.157* | 0.356** | 0.091 | 0.309** | 1 |  |  |
|  | KSGL | 0.043 | 0.125 | 0.146* | 0.096 | 0.157* | 0.012 | 0.118 | 0.125 | 0.050 | 0.130 | 0.112 | 0.117 | 1 |  |
|  | KSSVI | 0.092 | 0.194** | 0.163* | 0.079 | 0.060 | 0.206** | 0.257** | 0.196** | 0.291** | 0.144* | 0.294** | 0.793** | 0.690** | 1 |

Env, the specific environment. * and ** indicate significant levels at *P* < 0.05 and *P* < 0.01, respectively.
