# Supplementary material for: Identification of candidate tolerance genes to low-temperature during maize germination by GWAS and RNA-seqapproaches
Source: BMC Plant Biol. 2020 Jul 14;20:333. doi: 10.1186/s12870-020-02543-9 (PMC7362524; doi:10.1186/s12870-020-02543-9)
Supplement: Supplementary file 3 — Additional file 3 Figure S1. Estimated population structure of 222 maize inbred lines. [file 12870_2020_2543_MOESM3_ESM.docx]

**Additional file 3:**


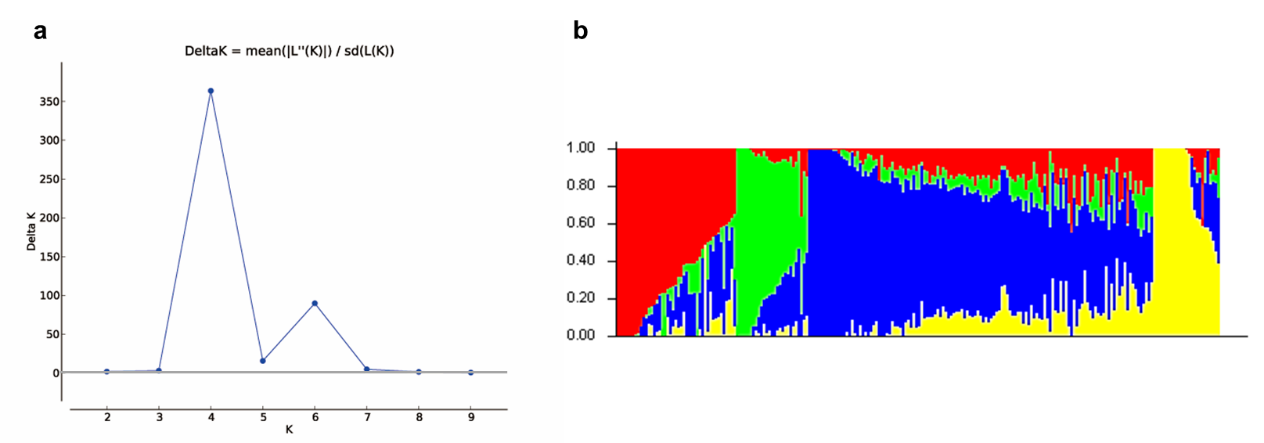


**Figure S1** Estimated population structure of 222 maize inbred lines. a. ∆K value of the determination. b. Estimated population structure of the 222 maize inbred lines with values of K = 4.
