# Supplementary material for: Identification of candidate tolerance genes to low-temperature during maize germination by GWAS and RNA-seqapproaches
Source: BMC Plant Biol. 2020 Jul 14;20:333. doi: 10.1186/s12870-020-02543-9 (PMC7362524; doi:10.1186/s12870-020-02543-9)
Supplement: Supplementary file 4 — Additional file 4 Table S3. Mean of LD decay distance of the 10 chromosomes for r2 values which are equal to greater than 0.1 and 0.2. [file 12870_2020_2543_MOESM4_ESM.docx]

**Additional file 4:**

**Table S3** Mean of LD decay distance of the 10 chromosomes for *r^2^* values which are equal to 0.1 and 0.2

| **Chromosome** | **LD decay(kb)** | |
| --- | --- | --- |
|  | ***r^2^* = 0.1** | ***r^2^* = 0.2** |
| **1** | 55 | 395 |
| **2** | 60 | 520 |
| **3** | 110 | 710 |
| **4** | 100 | 610 |
| **5** | 60 | 770 |
| **6** | 45 | 575 |
| **7** | 80 | 775 |
| **8** | 70 | 775 |
| **9** | 60 | 1125 |
| **10** | 110 | 850 |
| **Mean** | 60 | 710 |
