# Supplementary figures and images for: Identification of candidate tolerance genes to low-temperature during maize germination by GWAS and RNA-seqapproaches
Source: BMC Plant Biol. 2020 Jul 14;20:333. doi: 10.1186/s12870-020-02543-9 (PMC7362524; doi:10.1186/s12870-020-02543-9)

**Additional file 5:**


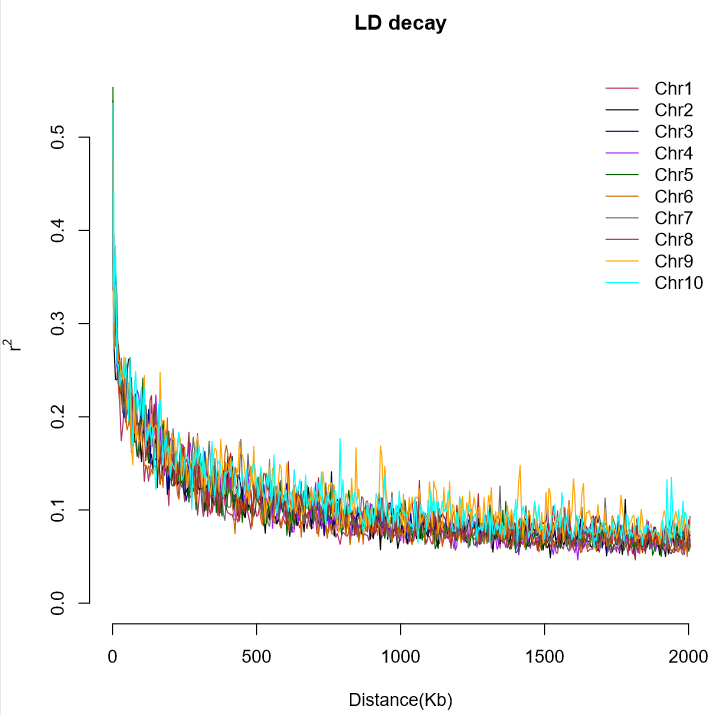


**Figure S2** Illustration of whole-genome LD in a panel of 222 maize lines.

Supplement: Supplementary file 5 — Additional file 5 Figure S2. Illustration of whole-genome LD in a panel of 222 maize lines. [file 12870_2020_2543_MOESM5_ESM.docx]
