# Supplementary material for: Identification of candidate tolerance genes to low-temperature during maize germination by GWAS and RNA-seqapproaches
Source: BMC Plant Biol. 2020 Jul 14;20:333. doi: 10.1186/s12870-020-02543-9 (PMC7362524; doi:10.1186/s12870-020-02543-9)
Supplement: Supplementary file 6 — Additional file 6 Table S4. Functions of an additional 68 candidate genes identified within a LD decay of 26 associated SNPs. [file 12870_2020_2543_MOESM6_ESM.docx]

**Additional file 6:**

**Table S4** Functions of an additional 68 candidate genes identified within a LD decay of 26 associated SNPs

| **SNP** | **MaizeGDB Gene ID** | **NCBI Gene ID** | **Gene function** |
| --- | --- | --- | --- |
| SYN25470 | *Zm00001d029190* | *LOC103634953* | very-long-chain 3-oxoacyl-CoA reductase-like protein At1g24470 |
|  | *Zm00001d029188* | *LOC100279862* | uncharacterized |
|  | *Zm00001d029189* | *LOC100192048* | R3H domain protein |
|  | *Zm00001d029191* | no | uncharacterized |
| PZE-101120376 | *Zm00001d029349* | *TIdP2891* | uncharacterized |
|  | *Zm00001d029347* | *LOC100279617* | uncharacterized |
| PZE-101192647 | *Zm00001d032915* | no | no |
|  | *Zm00001d032916* | *LOC103643550* | uncharacterized |
| PUT-163a-88747038-4526 | *Zm00001d034314* | *LOC103644121* | transcription initiation factor TFIId subunit 7 |
|  | *Zm00001d034312* | *LOC103644120* | proteoglycan 4-like |
|  | *Zm00001d034307* | *LOC103645664* | uncharacterized |
|  | *Zm00001d034318* | *cl35478_1d* | uncharacterized |
|  | *Zm00001d034319* | *LOC100282761* | inositolphosphorylceramide-B C-26 hydroxylase |
| PUT-163a-149007696-748 | *Zm00001d002244* | *LOC100382170* | Polyadenylate-binding protein RBP45C |
|  | *Zm00001d002240* | *CHL* | chloroplastic lipocalin |
|  | *Zm00001d002241* | *LOC100216700* | uncharacterized |
| SYN29778 | *Zm00001d002677* | *LOC107648858* | uncharacterized |
|  | *Zm00001d002675* | *LOC100284225* | anther-specific proline-rich protein APG |
| PZE-102100684 | *Zm00001d004633* | *LOC100193857* | Transmembrane amino acid transporter family protein |
| SYN21841 | *Zm00001d007340* | *LOC103649372* | AdP-ribosylation factor GTPase-activating protein AGd12 |
|  | *Zm00001d007341* | *si603006c09* | uncharacterized |
|  | *Zm00001d007343* | *LOC103648010* | HVA22-like protein e |
| PZE-103072583 | *Zm00001d041439* | *LOC103650480* | serine/threonine-protein kinase Nek2 |
| PZE-103094415 | *Zm00001d042226* | *no* | no |
|  | *Zm00001d042231* | *LOC100126948* | basal endosperm transfer layer10 |
| PZE-104024779 | *Zm00001d049443* | *ocl5* | outer cell layer5a |
|  | *Zm00001d049440* | *cl36914_1* | uncharacterized |
|  | *Zm00001d049441* | *LOC100193334* | Glucose-6-phosphate/phosphate translocator 2 |
| SYN30173 | *Zm00001d013416* | *LOC100384509* | PHd finger protein mALE mEIOCYTE dEATH 1 |
| PZE-105034900 | *Zm00001d013795* | *LOC100216568* | uncharacterized |
|  | *Zm00001d013796* | *LOC100192986* | dicer-like 105 |
|  | *Zm00001d013797* | *LOC103626078* | endoribonuclease dicer homolog 2a |
| PZE-106003222 | *Zm00001d035083* | *LOC103630843* | expansin-A6 |
| PZE-106097864 | *Zm00001d038371* | *lac2* | laccase-like |
| **SNP** | **maizeGdB Gene Id** | **NCBI Gene Id** | **Gene function** |
| PZE-106097864 | *Zm00001d038372* | *TIdP3268* | uncharacterized |
|  | *Zm00001d038373* | *LOC103630435* | uncharacterized |
| PZE-106129965 | *Zm00001d039219* | *si618049h06（54）* | uncharacterized |
|  | *Zm00001d039217* | *LOC100282776* | pepsin A |
| SYN4961 | *Zm00001d018862* | *LOC103631971* | pentatricopeptide repeat-containing protein At3g62890 |
|  | *Zm00001d018863* | *LOC103631972* | small RNA 2'-O-methyltransferase |
|  | *Zm00001d018864* | hda110 | histone deacetylase |
| PZE-107098845 | *Zm00001d021647* | *LOC103633239* | ABC transporter G family member 43 |
|  | *Zm00001d021652* | *LOC103633242* | 60S ribosomal protein L36a |
|  | *Zm00001d021653* | *LOC100281048* | glucose-6-phosphate/phosphate translocator 2 |
|  | *Zm00001d021654* | *LOC100276181* | uncharacterized LOC100276181 |
|  | *Zm00001d021655* | *LOC103633243* | WAT1-related protein At5g64700 |
| PUT-163a-78076151-4108 | *Zm00001d008219* | no | no |
|  | *Zm00001d008221* | *cl1316_1b* | cyclin5 |
|  | *Zm00001d008222* | *LOC100381542* | uncharacterized |
| PZE-108064544 | *Zm00001d010495* | *LOC100384528* | Vesicle-associated protein 2-2 |
|  | *Zm00001d010496* | *LOC100382875* | uncharacterized |
| PZE-108068725 | *Zm00001d010671* | *LOC100279614* | putative O-Glycosyl hydrolase superfamily protein |
|  | *Zm00001d010672* | *LOC100285605* | metacaspase type II |
| SYN26538 | *Zm00001d012511* | *pco102994b* | Protein kinase family protein with ARm repeat domain |
|  | *Zm00001d012512* | *LOC100384715* | uncharacterized |
|  | *Zm00001d012514* | *LOC100191484* | uncharacterized |
| PZE-109053558 | *Zm00001d046560* | *LOC103638716* | plasma membrane ATPase 4 |
|  | *Zm00001d046561* | *LOC100217243* | putative protein kinase superfamily protein |
| SYN5516 | *Zm00001d023537* | *pco098048* | uncharacterized |
|  | *Zm00001d023539* | *LOC100273197* | uncharacterized |
|  | *Zm00001d023540* | *GATA33* | uncharacterized |
| PZE-110057591 | *Zm00001d025255* | *LOC103641589* | cysteine-rich receptor-like protein kinase 25 |
|  | *Zm00001d025256* | *LOC109942894* | UdP-glycosyltransferase TURAN |
|  | *Zm00001d025258* | *LOC100280624* | dihydrolipoyllysine-residue succinyltransferase component of 2-oxoglutarate dehydrogenase complex |
| PZE-110060997 | *Zm00001d025379* | *LOC100284248* | photoperiod responsive protein |
|  | *Zm00001d025377* | *no* | no |
|  | *Zm00001d025383* | *LOC103641631* | G-type lectin S-receptor-like serine/threonine-protein kinase At2g19130 |
|  | *Zm00001d025382* | *LOC103641629* | histone H3.2 |
