# Supplementary material for: Identification of candidate tolerance genes to low-temperature during maize germination by GWAS and RNA-seqapproaches
Source: BMC Plant Biol. 2020 Jul 14;20:333. doi: 10.1186/s12870-020-02543-9 (PMC7362524; doi:10.1186/s12870-020-02543-9)
Supplement: Supplementary file 7 — Additional file 7 Table S5. Twenty-six GO terms associated with eight candidate genes. [file 12870_2020_2543_MOESM7_ESM.docx]

**Additional file 7:**

**Table S5** Twenty-six GO terms associated with eight candidate genes

| **Maize GDB Gene ID** | **GO_Term** | **GO_function** |
| --- | --- | --- |
| *Zm00001d021653* | carbohydrate transmembrane transport | biological_process |
| *Zm00001d034319* | fatty acid metabolic process | biological_process |
| *Zm00001d025379* | response to chitin | biological_process |
| *Zm00001d025379* | protein ubiquitination | biological_process |
| *Zm00001d038373* | fucose metabolic process | biological_process |
| *Zm00001d038373* | protein glycosylation | biological_process |
| *Zm00001d038373* | cell adhesion | biological_process |
| *Zm00001d038373* | cell wall organization | biological_process |
| *Zm00001d002677*; *Zm00001d029193* | biological_process | biological_process |
| *Zm00001d029193* | plant-type cell wall | cellular_component |
| *Zm00001d029193* | plasmodesma | cellular_component |
| *Zm00001d029193* | mitochondrion | cellular_component |
| *Zm00001d034319* | endoplasmic reticulum | cellular_component |
| *Zm00001d025379*; *Zm00001d002677* | cytoplasm | cellular_component |
| *Zm00001d038373* | Golgi apparatus | cellular_component |
| *Zm00001d038373; Zm00001d029193* | integral component of membrane | cellular_component |
| *Zm00001d038373* | Golgi membrane | cellular_component |
| *Zm00001d039219* | chloroplast | cellular_component |
| *Zm00001d039219*; *Zm00001d002676* | nucleus | cellular_component |
| *Zm00001d029193* | copper ion binding | molecular_function |
| *Zm00001d034319* | fatty acid alpha-hydroxylase activity | molecular_function |
| *Zm00001d025379* | ubiquitin-protein transferase activity | molecular_function |
| *Zm00001d038373* | transferase activity, transferring glycosyl groups | molecular_function |
| *Zm00001d029193* | oxidoreductase activity | molecular_function |
| *Zm00001d002677*; *Zm00001d029193* | molecular_function | molecular_function |
| *Zm00001d039219* | nucleoside diphosphate kinase activity | molecular_function |
