# Supplementary material for: Identification of candidate tolerance genes to low-temperature during maize germination by GWAS and RNA-seqapproaches
Source: BMC Plant Biol. 2020 Jul 14;20:333. doi: 10.1186/s12870-020-02543-9 (PMC7362524; doi:10.1186/s12870-020-02543-9)
Supplement: Supplementary file 8 — Additional file 8 Table S6. SNPs or candidate genes that overlap with published QTLs. [file 12870_2020_2543_MOESM8_ESM.docx]

**Additional file 8:**

**Table S6** SNPs or candidate genes that overlap with published QTLs

| **SNP** | **Bin** | **Reference** | **QTL or gene** | **Nearest marker** | | **Trait** |
| --- | --- | --- | --- | --- | --- | --- |
| PUT-163a-149007696-748 | 2.02 | Rodríguez et al. 2014 | QTL-8 | umc1823, umc1185 | | ФPSII |
| PZE-102099570 / PZE102100684 | 2.05 | Leipner et al. 2008 |  | dupssr21 | | straw dry weight |
|  |  | Fracheboud et al. 2004 |  | bnlg1909 | | *F′v*/*F′m* |
|  |  |  |  | bnlg1909 | | SPAD |
| PZE-108068725 | 8.04 | Li et al. 2018 | *Zm00001d010671 (GRMZM2G380561)* | |  | root length |
