# Supplementary material for: Identification of candidate tolerance genes to low-temperature during maize germination by GWAS and RNA-seqapproaches
Source: BMC Plant Biol. 2020 Jul 14;20:333. doi: 10.1186/s12870-020-02543-9 (PMC7362524; doi:10.1186/s12870-020-02543-9)
Supplement: Supplementary file 9 — Additional file 9 Table S7. Germination rates of 222 maize inbred lines at 25 °C. [file 12870_2020_2543_MOESM9_ESM.docx]

**Additional file 9:**

**Table S7** Germination rates of 222 maize inbred lines at 25 ℃

| **Code^a^** | **rep1^b^** | **rep2^c^** | **rep3^d^** | **GR^e^** |
| --- | --- | --- | --- | --- |
| 1 | 45 | 48 | 45 | 92.00% |
| 5 | 48 | 49 | 49 | 97.33% |
| 6 | 46 | 47 | 42 | 90.00% |
| 7 | 50 | 46 | 47 | 95.33% |
| 8 | 46 | 45 | 45 | 90.67% |
| 9 | 44 | 45 | 47 | 90.67% |
| 10 | 47 | 45 | 46 | 92.00% |
| 11 | 44 | 46 | 47 | 91.33% |
| 12 | 47 | 43 | 46 | 90.67% |
| 13 | 48 | 43 | 46 | 91.33% |
| 14 | 45 | 48 | 48 | 94.00% |
| 15 | 44 | 48 | 45 | 91.33% |
| 16 | 43 | 48 | 44 | 90.00% |
| 17 | 47 | 44 | 45 | 90.67% |
| 19 | 50 | 50 | 49 | 99.33% |
| 21 | 46 | 46 | 47 | 92.67% |
| 22 | 44 | 47 | 46 | 91.33% |
| 23 | 44 | 49 | 47 | 93.33% |
| 24 | 48 | 50 | 49 | 98.00% |
| 25 | 45 | 46 | 46 | 91.33% |
| 26 | 50 | 50 | 47 | 98.00% |
| 27 | 49 | 47 | 50 | 97.33% |
| 28 | 43 | 48 | 50 | 94.00% |
| 29 | 50 | 50 | 49 | 99.33% |
| 30 | 47 | 46 | 44 | 91.33% |
| 31 | 50 | 45 | 46 | 94.00% |
| 32 | 49 | 50 | 50 | 99.33% |
| 33 | 45 | 46 | 46 | 91.33% |
| 37 | 47 | 47 | 45 | 92.67% |
| 38 | 48 | 49 | 48 | 96.67% |
| 39 | 50 | 50 | 50 | 100.00% |
| 40 | 47 | 48 | 50 | 96.67% |
| 41 | 48 | 46 | 50 | 96.00% |
| 42 | 50 | 50 | 50 | 100.00% |
| 43 | 50 | 50 | 50 | 100.00% |
| 45 | 46 | 50 | 50 | 97.33% |
| 46 | 50 | 47 | 50 | 98.00% |
| 47 | 49 | 43 | 48 | 93.33% |
| 49 | 43 | 44 | 49 | 90.67% |
| 50 | 50 | 50 | 50 | 100.00% |
| **Code^a^** | **rep1^b^** | **rep2^c^** | **rep3^d^** | **GR^e^** |
| 51 | 48 | 48 | 49 | 96.67% |
| 52 | 44 | 47 | 44 | 90.00% |
| 53 | 47 | 46 | 44 | 91.33% |
| 55 | 49 | 49 | 50 | 98.67% |
| 56 | 48 | 48 | 49 | 96.67% |
| 57 | 50 | 50 | 47 | 98.00% |
| 58 | 49 | 48 | 48 | 96.67% |
| 59 | 45 | 45 | 45 | 90.00% |
| 60 | 49 | 46 | 46 | 94.00% |
| 61 | 50 | 50 | 49 | 99.33% |
| 62 | 49 | 46 | 50 | 96.67% |
| 63 | 44 | 49 | 46 | 92.67% |
| 64 | 43 | 47 | 47 | 91.33% |
| 65 | 49 | 47 | 44 | 93.33% |
| 66 | 45 | 43 | 49 | 91.33% |
| 67 | 44 | 46 | 45 | 90.00% |
| 68 | 44 | 45 | 46 | 90.00% |
| 69 | 48 | 47 | 45 | 93.33% |
| 70 | 50 | 47 | 45 | 94.67% |
| 71 | 45 | 49 | 43 | 91.33% |
| 72 | 50 | 50 | 50 | 100.00% |
| 73 | 48 | 50 | 48 | 97.33% |
| 74 | 47 | 47 | 47 | 94.00% |
| 75 | 49 | 47 | 49 | 96.67% |
| 77 | 45 | 42 | 48 | 90.00% |
| 78 | 44 | 49 | 45 | 92.00% |
| 79 | 48 | 48 | 50 | 97.33% |
| 80 | 42 | 47 | 47 | 90.67% |
| 81 | 47 | 49 | 48 | 96.00% |
| 82 | 48 | 44 | 49 | 94.00% |
| 83 | 45 | 47 | 45 | 91.33% |
| 85 | 50 | 48 | 49 | 98.00% |
| 87 | 48 | 49 | 46 | 95.33% |
| 89 | 47 | 47 | 46 | 93.33% |
| 90 | 45 | 48 | 43 | 90.67% |
| 91 | 42 | 50 | 46 | 92.00% |
| 92 | 45 | 47 | 44 | 90.67% |
| 94 | 49 | 49 | 50 | 98.67% |
| 95 | 47 | 47 | 46 | 93.33% |
| 96 | 49 | 42 | 44 | 90.00% |
| 97 | 50 | 47 | 50 | 98.00% |
| 98 | 45 | 45 | 46 | 90.67% |
| 101 | 46 | 47 | 46 | 92.67% |
| **Code^a^** | **rep1^b^** | **rep2^c^** | **rep3^d^** | **GR^e^** |
| 102 | 49 | 45 | 48 | 94.67% |
| 103 | 47 | 47 | 44 | 92.00% |
| 104 | 46 | 48 | 42 | 90.67% |
| 105 | 47 | 44 | 48 | 92.67% |
| 106 | 48 | 48 | 46 | 94.67% |
| 107 | 44 | 48 | 43 | 90.00% |
| 108 | 46 | 43 | 48 | 91.33% |
| 109 | 46 | 45 | 44 | 90.00% |
| 110 | 50 | 50 | 48 | 98.67% |
| 111 | 42 | 46 | 48 | 90.67% |
| 112 | 47 | 44 | 50 | 94.00% |
| 113 | 47 | 48 | 46 | 94.00% |
| 114 | 46 | 46 | 46 | 92.00% |
| 116 | 44 | 47 | 47 | 92.00% |
| 117 | 48 | 48 | 45 | 94.00% |
| 118 | 49 | 49 | 45 | 95.33% |
| 119 | 44 | 47 | 46 | 91.33% |
| 120 | 50 | 50 | 50 | 100.00% |
| 121 | 43 | 45 | 47 | 90.00% |
| 122 | 47 | 44 | 44 | 90.00% |
| 123 | 44 | 47 | 45 | 90.67% |
| 124 | 43 | 47 | 45 | 90.00% |
| 126 | 49 | 49 | 48 | 97.33% |
| 127 | 45 | 47 | 48 | 93.33% |
| 128 | 47 | 50 | 42 | 92.67% |
| 129 | 50 | 50 | 50 | 100.00% |
| 130 | 47 | 46 | 44 | 91.33% |
| 131 | 46 | 43 | 46 | 90.00% |
| 132 | 45 | 47 | 45 | 91.33% |
| 133 | 48 | 45 | 49 | 94.67% |
| 134 | 47 | 46 | 47 | 93.33% |
| 135 | 46 | 43 | 49 | 92.00% |
| 136 | 45 | 44 | 47 | 90.67% |
| 138 | 50 | 50 | 50 | 100.00% |
| 139 | 50 | 45 | 43 | 92.00% |
| 140 | 48 | 48 | 46 | 94.67% |
| 141 | 48 | 46 | 43 | 91.33% |
| 142 | 50 | 45 | 50 | 96.67% |
| 144 | 47 | 48 | 45 | 93.33% |
| 146 | 44 | 47 | 46 | 91.33% |
| 147 | 47 | 46 | 50 | 95.33% |
| 148 | 49 | 48 | 49 | 97.33% |
| 151 | 50 | 49 | 47 | 97.33% |
| **Code^a^** | **rep1^b^** | **rep2^c^** | **rep3^d^** | **GR^e^** |
| 153 | 48 | 50 | 47 | 96.67% |
| 154 | 44 | 47 | 44 | 90.00% |
| 156 | 50 | 43 | 47 | 93.33% |
| 157 | 48 | 49 | 44 | 94.00% |
| 158 | 48 | 48 | 47 | 95.33% |
| 159 | 47 | 48 | 48 | 95.33% |
| 160 | 46 | 47 | 47 | 93.33% |
| 161 | 46 | 46 | 48 | 93.33% |
| 162 | 45 | 50 | 48 | 95.33% |
| 163 | 49 | 45 | 45 | 92.67% |
| 164 | 50 | 50 | 50 | 100.00% |
| 165 | 50 | 48 | 48 | 97.33% |
| 166 | 45 | 45 | 47 | 91.33% |
| 167 | 50 | 50 | 50 | 100.00% |
| 169 | 50 | 48 | 47 | 96.67% |
| 170 | 50 | 47 | 48 | 96.67% |
| 171 | 50 | 48 | 48 | 97.33% |
| 172 | 47 | 48 | 47 | 94.67% |
| 173 | 48 | 48 | 48 | 96.00% |
| 174 | 50 | 50 | 50 | 100.00% |
| 175 | 48 | 48 | 50 | 97.33% |
| 176 | 48 | 46 | 47 | 94.00% |
| 179 | 49 | 49 | 47 | 96.67% |
| 180 | 49 | 50 | 49 | 98.67% |
| 181 | 47 | 48 | 47 | 94.67% |
| 182 | 48 | 47 | 47 | 94.67% |
| 183 | 48 | 44 | 44 | 90.67% |
| 184 | 49 | 48 | 49 | 97.33% |
| 185 | 50 | 50 | 50 | 100.00% |
| 188 | 47 | 44 | 50 | 94.00% |
| 189 | 43 | 48 | 46 | 91.33% |
| 190 | 46 | 48 | 50 | 96.00% |
| 192 | 49 | 50 | 48 | 98.00% |
| 194 | 47 | 44 | 46 | 91.33% |
| 195 | 46 | 45 | 44 | 90.00% |
| 196 | 43 | 48 | 46 | 91.33% |
| 198 | 48 | 50 | 45 | 95.33% |
| 200 | 48 | 43 | 47 | 92.00% |
| 201 | 44 | 47 | 46 | 91.33% |
| 204 | 50 | 50 | 48 | 98.67% |
| 205 | 48 | 47 | 47 | 94.67% |
| 206 | 49 | 49 | 49 | 98.00% |
| 207 | 47 | 49 | 45 | 94.00% |
| **Code^a^** | **rep1^b^** | **rep2^c^** | **rep3^d^** | **GR^e^** |
| 208 | 46 | 46 | 44 | 90.67% |
| 210 | 47 | 44 | 47 | 92.00% |
| 211 | 45 | 47 | 48 | 93.33% |
| 212 | 47 | 46 | 44 | 91.33% |
| 213 | 48 | 47 | 45 | 93.33% |
| 214 | 48 | 47 | 50 | 96.67% |
| 215 | 46 | 46 | 48 | 93.33% |
| 216 | 48 | 44 | 45 | 91.33% |
| 217 | 50 | 50 | 49 | 99.33% |
| 219 | 49 | 44 | 46 | 92.67% |
| 220 | 46 | 46 | 43 | 90.00% |
| 221 | 47 | 49 | 48 | 96.00% |
| 222 | 47 | 48 | 49 | 96.00% |
| 224 | 45 | 46 | 48 | 92.67% |
| 225 | 48 | 48 | 45 | 94.00% |
| 226 | 49 | 49 | 49 | 98.00% |
| 227 | 49 | 49 | 47 | 96.67% |
| 228 | 47 | 50 | 43 | 93.33% |
| 229 | 46 | 47 | 43 | 90.67% |
| 230 | 47 | 48 | 48 | 95.33% |
| 232 | 47 | 46 | 50 | 95.33% |
| 233 | 49 | 50 | 45 | 96.00% |
| 235 | 47 | 47 | 46 | 93.33% |
| 236 | 44 | 47 | 44 | 90.00% |
| 239 | 49 | 50 | 47 | 97.33% |
| 240 | 48 | 49 | 49 | 97.33% |
| 241 | 43 | 50 | 48 | 94.00% |
| 242 | 49 | 46 | 46 | 94.00% |
| 243 | 50 | 47 | 48 | 96.67% |
| 246 | 43 | 48 | 46 | 91.33% |
| 247 | 45 | 50 | 47 | 94.67% |
| 250 | 50 | 43 | 45 | 92.00% |
| 252 | 47 | 46 | 45 | 92.00% |
| 253 | 50 | 49 | 50 | 99.33% |
| 254 | 48 | 48 | 50 | 97.33% |
| 255 | 46 | 46 | 46 | 92.00% |
| 256 | 50 | 50 | 50 | 100.00% |
| 257 | 44 | 48 | 43 | 90.00% |
| 258 | 48 | 47 | 48 | 95.33% |
| 260 | 48 | 45 | 45 | 92.00% |
| 262 | 45 | 45 | 45 | 90.00% |
| 263 | 49 | 50 | 49 | 98.67% |
| 264 | 48 | 48 | 49 | 96.67% |
| **Code^a^** | **rep1^b^** | **rep2^c^** | **rep3^d^** | **GR^e^** |
| 265 | 49 | 49 | 50 | 98.67% |
| 267 | 46 | 47 | 47 | 93.33% |
| 269 | 46 | 48 | 48 | 94.67% |
| 271 | 50 | 49 | 50 | 99.33% |
| 272 | 50 | 50 | 48 | 98.67% |
| 273 | 49 | 50 | 45 | 96.00% |
| 278 | 47 | 45 | 49 | 94.00% |
| 279 | 49 | 46 | 45 | 93.33% |
| 280 | 46 | 47 | 49 | 94.67% |
| 283 | 47 | 45 | 46 | 92.00% |

^a^Codes of 222 maize inbred lines. ^b,c,d^Three replicates of germination numbers of maize seeds at 25 ℃. ^e^Average germination rate of three replicates. Each experimental replicate contained 50 seeds
