# Supplementary material for: Identification of candidate tolerance genes to low-temperature during maize germination by GWAS and RNA-seqapproaches
Source: BMC Plant Biol. 2020 Jul 14;20:333. doi: 10.1186/s12870-020-02543-9 (PMC7362524; doi:10.1186/s12870-020-02543-9)
Supplement: Supplementary file 10 — Additional file 10 Table S8. Primer sequences used for quantitative real-time PCR. [file 12870_2020_2543_MOESM10_ESM.docx]

**Additional file 10:**

**Table S8** Primer sequences used for quantitative real-time PCR

| **Gene name** | **Forward primer (5'-3')** | **Reverse primer (5'-3')** |
| --- | --- | --- |
| *Zm00001d039219* | AGAAACTTTACGAACCGAAATAGC | AAGCCAGTCATCCACATCAG |
| *Zm00001d029193* | CTCTGCTCTTGCTGCTCCTG | TGATGCCAATCACCTTCTGC |
| *Zm00001d002676* | CCAAATGAGCACAGCCGTAA | TTGGATGCCCTCAAGAC |
| *Zm00001d021653* | CTGTACCGTCAGGAGCATGT | AAGATAACGTTCAGCGCCCA |
| *Zm00001d034319* | CTGCCTGCTCGTGAAATCTA | ACCCGTGAAGAAGGTAATGC |
| *Zm00001d025379* | TGACGAGGCTGCTCCTCCT | GAGACAGCTCTTGGCGTTCACC |
